# HCV-Induced miR-21 Contributes to Evasion of Host Immune System by Targeting MyD88 and IRAK1

**DOI:** 10.1371/journal.ppat.1003248

**Published:** 2013-04-25

**Authors:** Yanni Chen, Junbo Chen, Hui Wang, Jingjing Shi, Kailang Wu, Shi Liu, Yingle Liu, Jianguo Wu

**Affiliations:** 1 State Key Laboratory of Virology, College of Life Sciences, and Chinese-French Liver Disease Research Institute at Zhongnan Hospital, Wuhan University, Wuhan, Hubei, People′s Republic of China; 2 State Key Laboratory of Virology, Wuhan Institution of Virology, Chinese Academy of Sciences, Wuhan, Hubei, People′s Republic of China; 3 Wuhan Institute of Biotechnology, Wuhan East Lake High Technology Development Zone, Wuhan, Hubei, People′s Republic of China; The Rockefeller University, United States of America

## Abstract

Upon recognition of viral components by pattern recognition receptors, such as the toll-like receptors (TLRs) and retinoic acid-inducible gene I (RIG-I)-like helicases, cells are activated to produce type I interferon (IFN) and proinflammatory cytokines. These pathways are tightly regulated by the host to prevent an inappropriate cellular response, but viruses can modulate these pathways to proliferate and spread. In this study, we revealed a novel mechanism in which hepatitis C virus (HCV) evades the immune surveillance system to proliferate by activating microRNA-21 (miR-21). We demonstrated that HCV infection upregulates miR-21, which in turn suppresses HCV-triggered type I IFN production, thus promoting HCV replication. Furthermore, we demonstrated that miR-21 targets two important factors in the TLR signaling pathway, myeloid differentiation factor 88 (MyD88) and interleukin-1 receptor-associated kinase 1 (IRAK1), which are involved in HCV-induced type I IFN production. HCV-mediated activation of miR-21 expression requires viral proteins and several signaling components. Moreover, we identified a transcription factor, activating protein-1 (AP-1), which is partly responsible for miR-21 induction in response to HCV infection through PKCε/JNK/c-Jun and PKCα/ERK/c-Fos cascades. Taken together, our results indicate that miR-21 is upregulated during HCV infection and negatively regulates IFN-α signaling through MyD88 and IRAK1 and may be a potential therapeutic target for antiviral intervention.

## Author Summary

Hepatitis C virus (HCV), a major cause of chronic hepatitis, end-stage cirrhosis, and hepatocellular carcinoma, has chronically infected 200 million people worldwide and 3–4 million more each year. When triggered by viral infection, host cells produce type I interferon (IFN) and proinflammatory cytokines to antagonize the virus. Despite extensive research, the mechanism underlying HCV immune system evasion remains elusive. Our results provided the first direct evidence that microRNA-21 (miR-21) feedback inhibits type I IFN signaling when cells are challenged with HCV, thus promoting the infection. MicroRNA is a kind of endogenous non-coding small RNA that regulates a wide range of biological processes and participate in innate and adaptive immune responses through complementarily pairing with target mRNA, which can regulate its expression or translation. Currently, miRNAs have intrigued many scientists as potent targets or therapeutic agents for diseases. In our study, the targets of miR-21, myeloid differentiation factor 88 (MyD88) and interleukin-1 receptor-associated kinase 1 (IRAK1), which are important for HCV-induced type I IFN production, have also been found. Moreover, we identified a transcription factor, AP-1, which is partly responsible for miR-21 induction in response to HCV infection. Taken together, our research has provided new insights into understanding the effects of miRNA on host-virus interactions, and revealed a potential therapeutic target for antiviral intervention.

## Introduction

The hepatitis C virus (HCV) is a small, enveloped positive-sense RNA virus of the Flaviviridae family (Hepacivirus genus) [1], [2]. Since its discovery in 1989 [3], HCV has been revealed as a primary cause of chronic hepatitis, end-stage cirrhosis, and hepatocellular carcinoma (HCC). Worldwide, an estimated 200 million persons (approximately 3% of the global population) are chronically infected with HCV, and 3 to 4 million persons are newly infected each year [4], [5]. Naturally occurring variants of HCV are classified into six major genotypes. The 9.6-kb HCV genome encodes a large polyprotein that is processed by viral and cellular proteins to produce the virion structural proteins (core protein and glycoproteins E1 and E2) and nonstructural (NS) proteins (p7, NS2, NS3, NS4A, NS4B, NS5A, and NS5B) [3], [4].

Innate and adaptive antiviral immune responses are essential for host survival during viral infection. Upon recognition of viral components, host cells are activated to produce type I interferon (IFN) and pro-inflammatory cytokines, thereby upregulating a family of IFN-stimulated genes (ISGs) that exert pleiotropic inhibitory effects on viral replication in neighboring cells [6], [7]. Type I IFN production requires tight control to achieve the appropriate immune response to invading pathogens without triggering an immune disorder [8]. Consequently, viruses have developed strategies to evade and antagonize the host immune response and resist the antiviral actions of IFN therapy. However, the mechanism(s) underlying such immune evasion is not clear.

MicroRNAs (miRNAs) are an abundant class of highly conserved small non-coding RNAs. They function primarily by binding to the 3′ untranslated region (UTR) of target mRNAs to achieve post-transcriptional regulation of gene expression [9]. Many miRNAs have been reported to regulate a wide range of biological processes, including development [10], cell differentiation [11], proliferation, and apoptosis [12]–[14]. Many miRNAs, including miR-146 [15], miR-155 [16]–[18], miR-98, and let-7 [19], participate in innate and adaptive immune responses [20]–[22]. miR-21 was found to be more strongly expressed in HCC specimens than in non-tumorous tissues [23], and participates in HCC development by regulating the phosphatase and tensin homolog deleted on chromosome ten (PTEN) gene [24]. miR-21 is also overexpressed in HCV-positive liver biopsy samples, as assessed by microarray analysis [25]. Although these results suggest the involvement of miR-21 in antiviral responses, no studies have reported the mechanism that underlies the regulation of type I IFN signaling mediated by miR-21 or the antiviral response of miR-21 to type I IFN signaling.

In the present study, we analyzed the miR-21 expression profile in human hepatocytes during HCV infection and found that miR-21 was rapidly upregulated following HCV infection. Upregulated miR-21 suppressed MyD88 and IRAK1 expression in hepatocytes, which subsequently repressed type I IFN effector gene expression and the type I IFN-mediated antiviral response, thereby promoting viral replication. In addition, miR-21 also enhanced the replication of other viruses such as enterovirus 71 (EV71), human immunodeficiency virus (HIV), and vesicular stomatitis virus (VSV) by repressing type-I IFN production. To the best of our knowledge, this report is the first to show that miR-21 is upregulated upon RNA virus infection and acts as a negative regulator of type I IFN signaling by targeting MyD88 and IRAK1.

## Results

### HCV infection upregulates miR-21 expression

**Figure 1:**
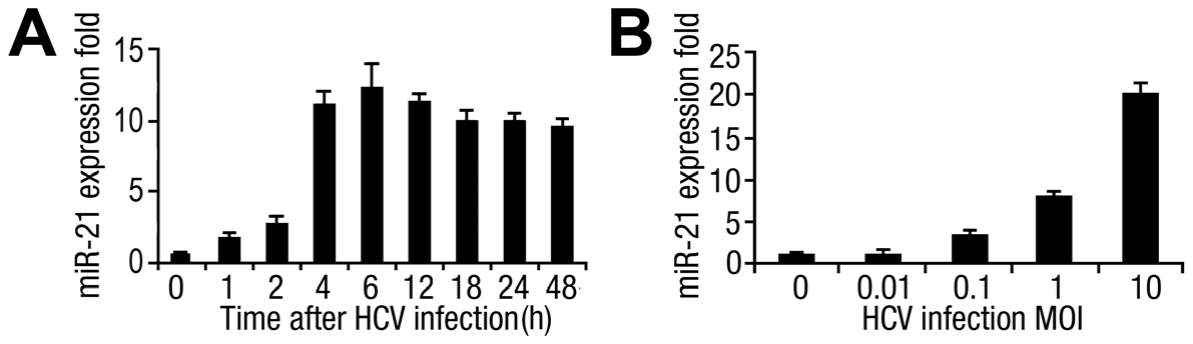
Determination of the expression of miR-21 during HCV infection in hepatocytes. (*A*) Human Huh7 hepatocytes were infected with or without HCV (MOI = 1) for different times as indicated. The expression of miR-21 was measured by qPCR and normalized to the expression of U6 in each sample. Results are standardized to 1 in uninfected cells. (*B*) Human Huh7 hepatocytes were infected with or without HCV at different MOIs, as indicated, for 12 h, and expression miR-21 was determined by qPCR. Data are presented as the means
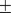
SD (n = 3) from one representative experiment. Similar results were obtained in three independent experiments.

Although miR-21 is highly expressed in HCC [24] and overexpressed in HCV-positive liver biopsy specimens, as assessed by microarray analysis [23], [25], the mechanism involved in the regulation of miR-21 by HCV infection is still unknown. To determine the relationship between HCV infection and miR-21 activation, we determined the levels of miR-21 in hepatocytes infected with the virus. The results from quantitative polymerase chain reaction (qPCR) analysis showed that miR-21 levels were increased in human Huh7 hepatocytes 1 h after infection HCV strain JFH-1 and reached a peak 6 h after HCV exposure (Figure 1A); this response was dose-dependent (Figure 1B). To understand the kinetics of miR-21 induction in HCV infection, we measured the expression of primary miR-21 (pri-miR-21) and miR-21 precursor (pre-miR-21). The induction of pri-miR-21 and pre-miR-21 were coincided with that of mature miR-21 and occurred as early as 2h.p.i. (Figure S1A). We cannot rule out the possibility that the increased miR-21 induced by HCV infection was due to nonspecific effects. To assess this possibility, a random non-HCV-related miRNA (miR-93) was analyzed after HCV infection (Figure S1B). Moreover, cells inoculated with UV-irradiated inactive HCV were also used as another negative control. As the same result of miR-93, the expression level of miR-21 was shown to have no significant changes (Figure S1C). These results indicated that HCV activated miR-21 expression in hepatocytes.

To confirm the above results, the effect of a small interfering RNA directly targeting HCV (si-HCV) on the HCV-regulated induction of miR-21 was examined. Huh7 cells were transfected with pFL-J6/JFH5′C19Rluc2Aubi RNA and si-HCV or control siRNA (si-Ctrl). The results showed that Renilla luciferase activity was inhibited by treatment with si-HCV, but not with si-Ctrl (Figure S1D), indicating that HCV replication was inhibited by si-HCV. The results from qPCR showed that miR-21 levels were also significantly reduced in the presence of si-HCV (Figure S1E), suggesting that HCV activated miR-21 expression.

### The HCV NS3/4A and NS5A proteins activate miR-21 expression through the c-Fos and c-Jun signaling proteins

**Figure 2:**
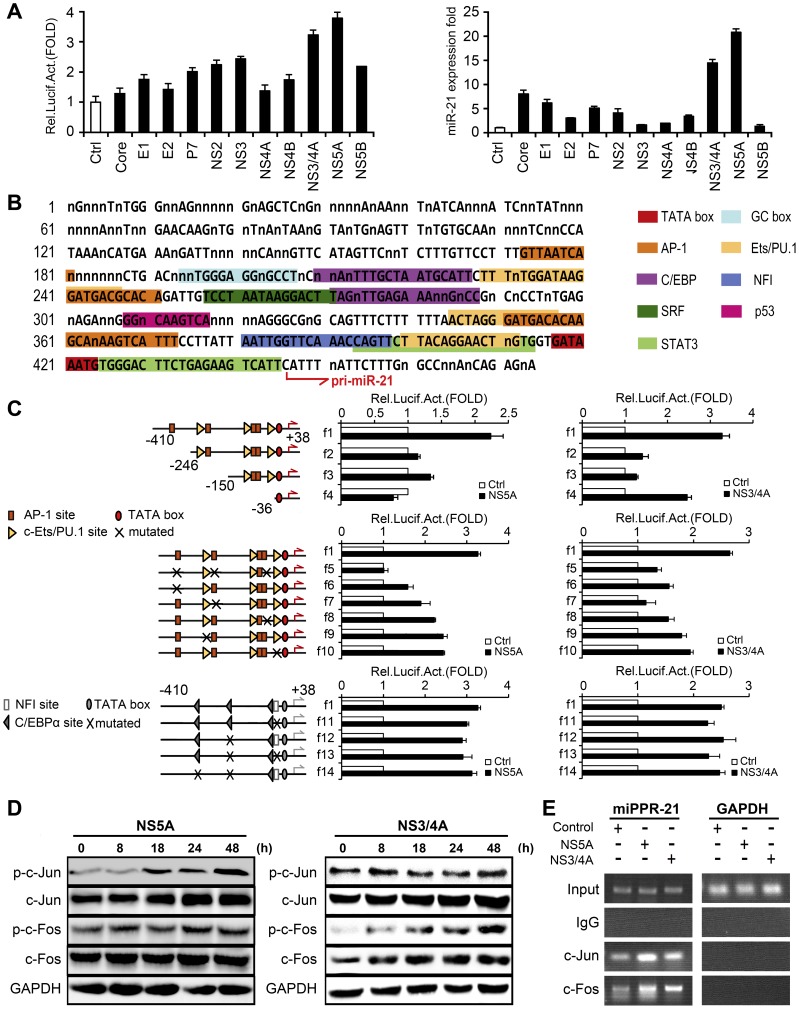
Functional analysis of *cis*-regulatory elements involved in the activation of miR-21 regulated by HCV proteins. (A) Huh7 cells were co-transfected with miR-21-luciferase reporter plasmid (miPPR-21) and the plasmids encoding each of the 11 HCV proteins, as indicated, for 24 h. The activity of the miR-21 promoter was measured by luciferase activity assays (*left panel*), and the levels of miR-21 expression were determined by qPCR (*right panel*). (B) The consensus sequences of the miR-21 promoter region (−410 to +38) in the reporter plasmid miPPR-21. Conserved bases across vertebrates are shown in capitals and nonconserved bases or deletions are denoted by “n”. The potential *cis*-acting elements for transcription factors are indicated. (C) Promoter analysis by transient expression of a series of reporter plasmids of miPPR-21 (intact, f1; truncated, f2–f4; mutated, f5–f14) in Huh7 cells. Data are presented as the means
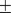
SD from three experiments. (D) Huh7 cells were transfected with pCMV-NS5A or pCMV-NS3/4A at different time intervals, respectively. The phosphorylation and total protein levels of c-Jun and c-Fos were determined by Western blot. (E) AP-1 binding sites were determined by ChIP assays. All experiments were repeated at least three times with similar results.

Because HCV is involved in the regulation of miR-21 expression, we next wanted to determine which, if any, of the HCV proteins was responsible for such regulation. Huh7 cells were co-transfected with plasmids expressing each of the 11 HCV proteins and miPPR21, which containing the miR-21 promoter spanning the −410 to +38 bp region. Luciferase activity assay indicated that the NS3/4A complex and NS5A protein, exert the most stimulatory effects on the activation of the miR-21 promoter (Figure 2A, left panel). qPCR analysis also showed that the NS3/4A complex and NS5A protein can enhance the expression of miR-21 (Figure 2A, right panel).

Several conserved regulatory elements that have previously been reported [26] were found in the consensus sequence of miPPR-21 (Figure 2B), including binding sites for activation protein 1 (AP-1), an E-twenty six family transcription factor (Ets/PU.1), CCAAT/enhancer binding protein α (C/EBPα), nuclear factor I (NFI), signal recognition particle (SRP), the p53 transcription factor (p53), and signal transducer and activator of transcription 3 (STAT3). To analyze the roles of these regulatory elements in the regulation of HCV-mediated miR-21 expression, we constructed a series of reporter plasmids (f1–f4) in which either full-length miPPR-21 or truncation mutants of this promoter were fused to the 5′ end of the firefly luciferase gene (Figure 2C, upper panel). Full-length miPPR-21 displays clear NS5A (Figure 2C, right panel) or NS3/4A (Figure 2C, left panel) inducibility, which is decreased by truncations of this promoter region, indicating that the distal AP-1 site is involved in the induction of miR-21 by NS5A and NS3/4A. Similar results were obtained from a different experiment showing that AP-1 was involved in the activities of HCV NS5A and NS3/4A proteins (Figure S2).

It has been well established that AP-1 functions synergistically with members of the Ets/PU.1 family. Experiments using another set of reporters, where several binding sites for AP-1 or Ets/PU.1 were mutated (f5–f10) indicated that the AP-1 elements in miPPR-21 are crucial for its NS5A and NS3/4A inducibility (Figure 2C middle panel). In addition, some mutations in the NFI and C/EBPα binding sites (f11–f14) in miPPR-21 showed no significant effects (Figure 2C lower panel).

Because AP-1 consists of two subunits, c-Jun and c-Fos, it was necessary to find out which subunit of AP-1 is responsible for the HCV-mediated activation of miPPR-21. Western blot analysis was used to determine the expression levels of c-Jun and c-Fos as regulated by NS5A and NS3/4A, respectively. The levels of both phosphorylated and total c-Jun, but not c-Fos, were activated by NS5A in a time-dependent fashion (Figure 2D, left panel). In addition, the levels of phosphorylated and total c-Fos, but not c-Jun, were stimulated by the NS3/4A complex in a time-dependent fashion (Figure 2D, right panel). These results suggested that the NS5A and NS3/4A proteins may stimulate the miR-21 expression through the same AP-1 element site, but the specific protein involved in this pathway may be different.

In addition, a chromatin immunoprecipitation (ChIP) assay was performed to confirm that the binding of c-Jun to the miPPR-21 promoter was enhanced by the NS5A protein, and the binding of c-Fos to miPPR-21 promoter was stimulated by NS3/4A complex (Figure 2E, left panel). In contrast, the promoter region of a HCV-noninducible housekeeping gene, GAPDH, was not coimmunoprecipitated by any of these factors (Figure 2E, right panel). Together these results demonstrated that both NS5A and NS3/4A proteins of HCV stimulate miR-21 expression through AP-1, but NS5A regulates miR-21 mainly through c-Jun and NS3/4A mediates miR-21 mainly through c-Fos.

### PKC-JNK and PKC-ERK signaling pathways are involved in the activation of miR-21 regulated by HCV NS5A and NS3/4A proteins

It is well known that members of the mitogen-activated protein kinase (MAPK) family regulate transcription factors that are important in the regulation of AP-1 activation and expression, which has been shown to depend on different kinase activators in various cell types. Based on our finding that NS5A-regulated activation and NS3/4A-regulated activation of miR-21 required c-Jun and c-Fos, respectively, we investigated the roles of kinases and the molecular mechanisms underlying this event.

**Figure 3:**
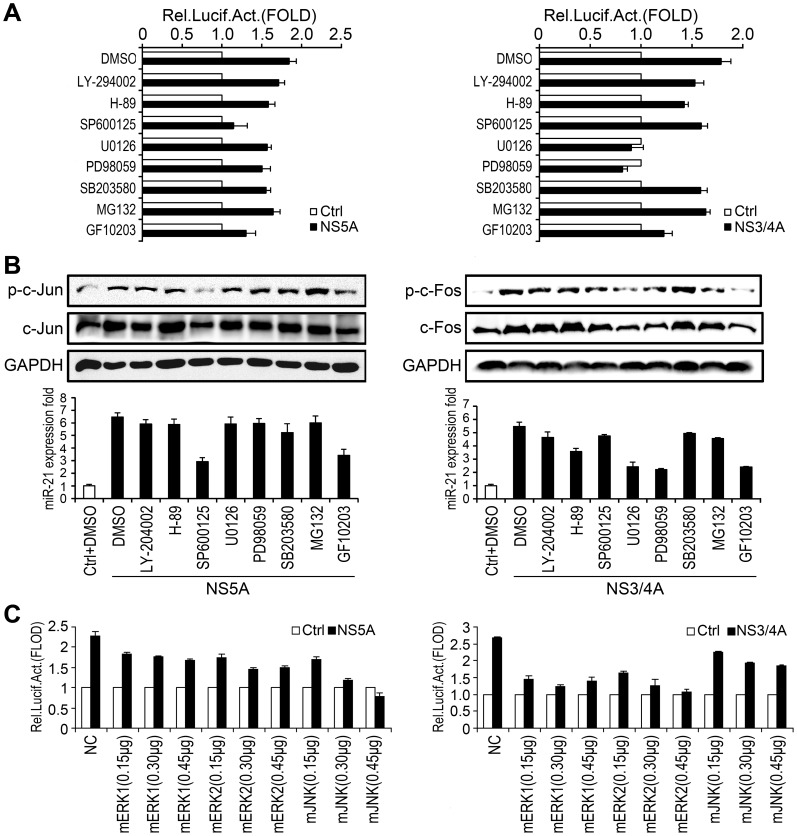
Investigation of the roles of ERK, JNK, and PKC in the regulation of miR-21 expression upon HCV infection. (A) Huh7 cells were co-transfected with miPPR21 and pCMV-NS5A (*left panel*) or pCMV-NS3/4A (*right panel*) for 24 h, and then signal pathway specific inhibitors (20 µM each) were then added, as indicated. The cells were lysed and luciferase activity was measured. (B) Cells were transfected with pCMV-NS5A (*left panel*) or pCMV-NS3/4A (right panel) for 24 h, and then treated with the signal pathway inhibitors (20 µM each) as indicated. The phosphorylation and total protein levels of c-Jun (*left panel*) and c-Fos (*right panel*) were determined by Western blot (*upper panel*), and miR-21 expression was measured by qPCR (*lower panel*). (C) Huh7 cells were co-transfected with miPPR21 and dominant-negative mutants of ERK1 (mERK1), ERK2 (mERK2), JNK (mJNK) or control vectors at different concentrations, as indicated and the resultant luciferase activities were measured. All experiments were repeated at least three times with similar results. Bar graphs represent the means ± SD, n = 3.

Cells co-transfected with the reporter plasmid miPPR-21 and pCMV-NS5A or pCMV-NS3/4A were treated with inhibitors of different signaling pathway components, including LY-294002 (Phosphoinostitde 3-Kinase (PI3K)-specific inhibitor), H-89 (cAMP-dependent protein kinase (PKA)-specific inhibitor), SP600125 (Jun N-terminal kinase (JNK)-specific inhibitor), U0126 (MAPK/ERK kinase (MEK)/extracellular signal regulated kinase (ERK)-specific inhibitor), PD98059 (ERK-specific inhibitor), SB203580 (P38 MAP kinase-specific inhibitor), MG132 (nuclear factor-κB (NF-κB) specific inhibitor), and GF109203 (protein kinase C (PKC)-specific inhibitor). The results from luciferase activity assays showed that the miR-21 promoter activity induced by NS5A decreased significantly in the presence of SP600125 and GF10203 (Figure 3A, left panel). Additionally, the miR-21 promoter activity up-regulated by NS3/4A was clearly repressed by U0126, PD98059, and GF10203 (Figure 3A, right panel). Western blot and qPCR analyses revealed a reduction of the phosphorylation and total protein levels of c-Jun or c-Fos with a corresponding decrease in the expression level of miR-21 stimulated by NS5A (Figure 3B, left panel) or NS3/4A (Figure 3B, right panel), respectively.

The roles of ERK and JNK in the NS5A- and NS3/4A-mediated activation of miR-21 were further examined using three dominant kinase-inactive mutants of ERK or JNK (mERK1, mERK2, and mJNK), the expression of which can block kinase activities by competing with endogenous kinases. Cells were co-transfected with pCMV-NS5A or pCMV-NS3/4A, miPPR-21, and each of the three kinase mutants. Reporter gene expression, measured as luciferase activity, was reduced by the JNK or ERK mutants in cells transfected with NS5A (Figure 3C, left panel) or NS3/4A (Figure 3C, right panel), respectively, and this response was dose-dependent. Taken together, these data suggested that ERK, JNK and PKC are involved in HCV-induced miR-21 activation, while the NS5A protein may specifically trigger the PKC-JNK pathway and NS3/4A may trigger the PKC-ERK pathway.

### NS5A- and NS3/4A-stimulated the phosphorylation of JNK and ERK1/2 results in the binding of AP-1 to the miR-21 promoter via the PKCε and PKCα signal transduction pathways

**Figure 4:**
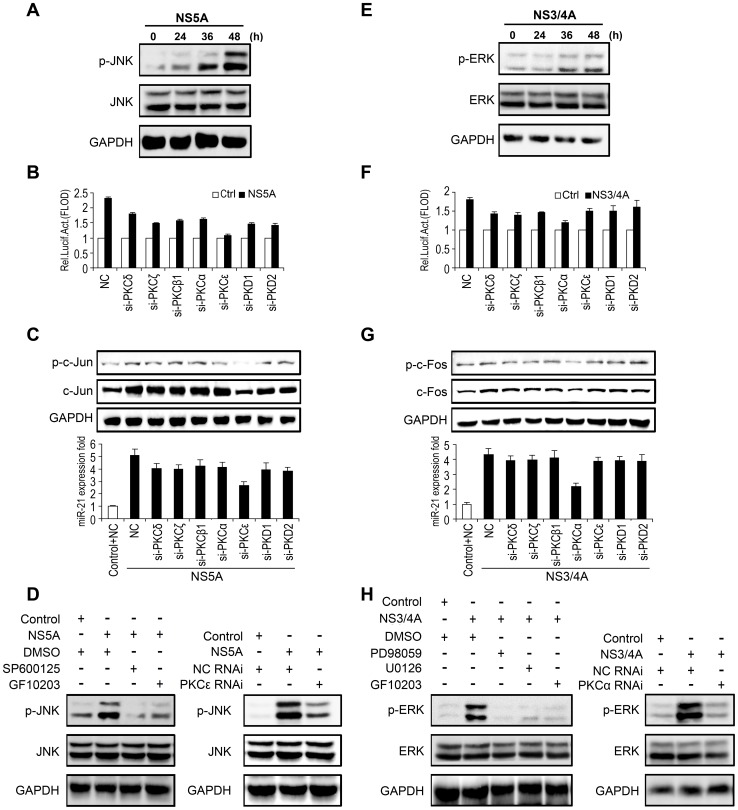
Involvement of PKCε and PKCα in the induction of miR-21. (*A and E*) Time-dependent JNK (*A*) and ERK (*E*) phosphorylation determined by Western blot. (*B and F*) Huh7 cells were co-transfected with miPPR-21 and pCMV-NS5A (*B*) or pCMV-NS3/4A (*F*) along with siRNA expression plasmids against different isoforms of PKC or siCtrl. A similar vector (pSilencer 2.0) containing an irrelevant sequence that does not show significant homology to any human gene was provided by Ambion, Inc. and used as a negative control. Luciferase activities were measured. (*C and G*) Cells were transfected with pCMV-NS3/4A (*C*) or pCMV-NS5A (*G*) and treated with PKC siRNA expression plasmids or si-Ctrl, as indicated. The phosphorylation and total protein levels of c-Jun (*C*) and c-Fos (*G*) were determined by Western blot (upper panel), and miR-21 expression was measured by qPCR (lower paned). (*D*) Inhibition of p-JNK by SP600125, GF109203, and PKCε-specific siRNA (PKCε siRNA) in the presence of NS3/4A presented. (*H*) U0126, GF109203, and PKCα-specific siRNA (PKCα siRNA) attenuated ERK phosphorylation when treated with NS5A. All experiments were repeated at least three times with similar results. Bar graphs represent means ± SD, n = 3.

To further investigate the role of the two HCV proteins in the activation of signal transduction pathways, we determined the phosphorylation status of JNK and ERK1/2 in Huh7 cells via Western blot analysis using antibodies specifically recognizing phosphorylated JNK and ERK1/2. The results showed that the levels of phosphorylated JNK or phosphorylated ERK1/2 increased in a time-dependent fashion in the presence of NS5A (Figure 4A) or NS3/4A protein (Figure 4E), respectively. Phosphorylation of JNK was blocked by both SP600125 and GF109203 (Figure 4D, left panel). Accordingly, the level of ERK1/2 phosphorylation was significantly reduced by PD98059, U0126, and GF109203 (Figure 4H, left panel). These results indicated that the JNK and ERK phosphorylation induced by the two HCV proteins is dependent on the PKC transduction pathway.

The PKC family has been shown to be involved in signal transduction associated with a wide range of biological responses, triggering changes in cell morphology, proliferation, and differentiation [27], [28]. As NS5A and NS3/4A-activated ERK and JNK were dependent on PKC, we extended our study to identify the specific isozymes of PKC involved in the HCV-regulated activation of miR-21 using an RNA interference (RNAi) approach. Molecules of small interference RNA (siRNA) that specifically knock down genes encoding each PKC isozyme were designed and used to study the function of PKC, based on a previous study [29]. We previously demonstrated that these siRNA molecules were specific and effective [30], [31]. Our results indicated that PKCε-specific and PKCα-specific siRNA (PKCε RNAi and PKCα RNAi, respectively) were able to significantly inhibit the induction of the miR-21 promoter in cells transiently co-transfected with NS5A (Figure 4B) or NS3/4A (Figure 4F), respectively. Western blot analyses were used to detect the phosphorylation or total protein levels of c-Jun or c-Fos induced by NS5A (Figure 4C, upper panel) or NS3/4A (Figure 4G, upper panel), respectively. The miR-21 expression levels stimulated by these two proteins were measured by qPCR (Figure 4C and 4G, lower panel). These results confirmed the reduction seen in the corresponding luciferase reporter assays.

The effects of PKCε and PKCα on the phosphorylation of JNK and ERK were also evaluated using another RNAi approach. The results from the Western blot analysis showed that phosphorylated JNK levels stimulated by the NS5A protein were significantly decreased upon treatment with PKCε-specific siRNA (PKCε RNAi) (Figure 4D, right panel). Similarly, the phosphorylation of ERK1/2 activated by the NS3/4A protein was inhibited by PKCα-specific siRNA (PKCα RNAi) (Figure 4H, right panel). All of these results suggested that PKCε is the upstream kinase involved in NS5A-induced JNK/c-Jun activation, and PKCα is the upstream kinase in NS3/4A induced ERK/c-Fos activation.

### miR-21 suppresses HCV-triggered type I IFN production in hepatocytes

It is known that miRNAs regulate a wide range of biological processes, including development, cell differentiation, proliferation, and apoptosis. Some miRNAs, including miR-146, miR-155, miR-98, and let-7, participate in the innate and adaptive immune responses. Although our results demonstrated that miR-21 was activated by HCV proteins through two signaling pathways, the biological effects of miR-21 have not previously been studied. Thus, we investigated the role of miR-21 in the regulation of innate and adaptive immune responses during HCV infection.

**Figure 5:**
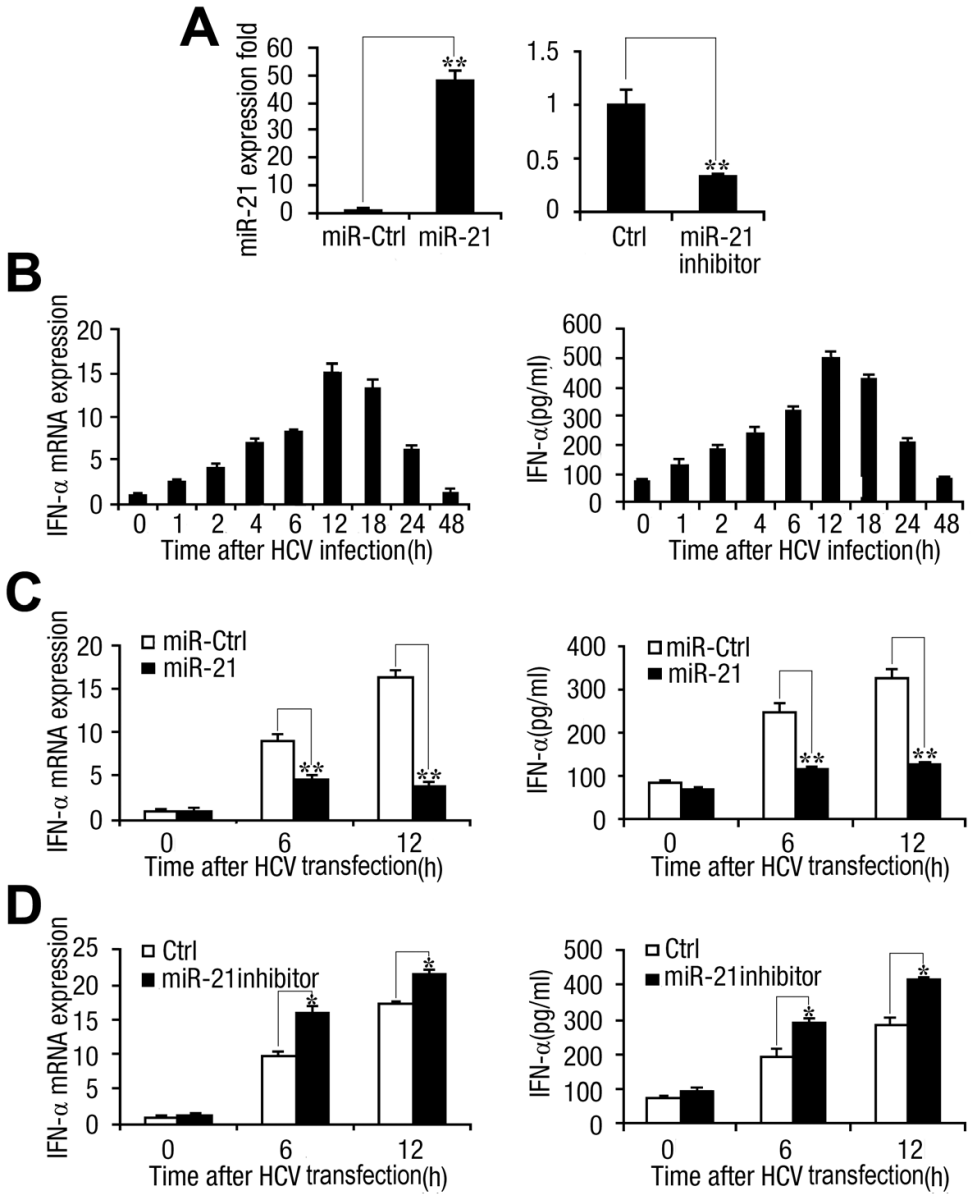
miR-21 suppresses HCV-triggered type I IFN production. (A) Human Huh7 hepatocytes (0.5 ml, 2×10^5^ cells) were transfected with control miRNA (miR-ctrl) or miR-21 mimics (*left*), and control inhibitor (ctrl) or miR-21 inhibitor (*right*), as indicated at a final concentration of 50 nM. RNA was isolated 48 h post-transfection, and miR-21 expression was measured by qPCR and normalized to U6 snRNA. Results are standardized to 1 in control cells. (B) Huh7 hepatocytes were infected with or without HCV (MOI = 1) for different times as indicated. IFN-α mRNA levels (*left*) were determined by qPCR and normalized to the expression of GAPDH in each sample. IFN-α secretion into the cell culture medium (*right*) was measured by ELISA. (*C* and *D*) Huh7 hepatocytes were transfected with miR-21 mimics or control RNA (*C*), miR-21 inhibitor or control inhibitor (*D*), as indicated. After 48 h, the cells were transfected with FL-J6/JFH5′C19Rluc2AUbi (0.1 µg) for the indicated time. IFN-α mRNA levels were determined by qPCR, and IFN-α secretion into the medium was measured by ELISA. Data are shown as the means
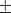
SD (n = 3) from one representative experiment. Similar results were obtained in three independent experiments. **, p<0.01; *, p<0.05.

First, the effects of synthetic miR-21 mimics and miR-21 inhibitor on the expression of miR-21 were evaluated. miR-21 inhibitor is a synthetic oligonucleotide with exact sequence complementarity to miR-21. Such inhibitor oligonucleotides have been shown to sequester intracellular miRNAs and to inhibit their activity in the RNA-interfering pathway [32]. Huh7 cells were transfected with miR-21 or nonspecific control RNA oligonucleotides (miR-Ctrl) and miR-21 inhibitor or Ctrl-inhibitor, respectively. As expected, the miR-21 mimics increased miR-21 expression significantly in Huh7 hepatocytes, whereas miR-21 inhibitors decreased miR-21 expression (Figure 5A). To determine whether HCV infection stimulates IFN-α expression, Huh7 cells were infected with the JFH-1 HCV strain at a high multiplicity of infection (MOI = 1), and IFN-α expression was monitored. Time course analysis revealed that IFN-α was rapidly induced, reaching peak concentrations within 12 h (Figure 5B). However, the levels of IFN-α mRNA and protein were not significantly affected in Huh7 cells inoculated with UV-inactivated HCV (Figure S3A).

As a comparison, we also examined the effects of transfection of the pFL-J6/JFH5′C19Rluc2AUbi on the induction of miR-21 and IFN-α in Huh7 cells. The results revealed that the levels of miR-21 (Figure S3B, left panel) and IFN-α (Figure S3B, right panel) rapidly increased in Huh7 cells transfected with pFL-J6/JFH5′C19Rluc2AUbi and reached a peak at 12 h post-transfection (Figure S3B). The ability of the plasmids to replicated HCV infection was confirmed by Renilla luciferase activity assay (Figure S3C). These results demonstrated that the effects of pFL-J6/JFH5′C19Rluc2AUbi on the induction of miR-21 and IFN-α were equivalent to that of the JFH-1 HCV strain. To ensure HCV production, FL-J6/JFH5′C19Rluc2AUbi transfection was primarily used in the subsequent experiments, which required short investigation time.

The effects of miR-21 and HCV on the expression of IFN-α were then determined. Huh7 cells were infected with the JFH-1 HCV strain and treated with miR-21, miR-Ctrl, miR-21 inhibitor, and Ctrl-inhibitor, respectively. Our results indicated that miR-21 overexpression reduced IFN-α mRNA and protein levels in HCV-exposed cells (Figure 5C), whereas miR-21 inhibitors caused a modest but reproducible enhancement of IFN-α expression (Figure 5D). These results demonstrated that HCV infection activates miR-21 expression and miR-21 suppresses the production of type I IFN.

We further investigated the effect of miR-21 on HCV induction of pro-inflammatory cytokines and chemokines in Huh7 hepatocytes. Huh7 cells were transfected with pFL-J6/JFH5′C19Rluc2Aubi and treated with miR-21 or miR-Ctrl. Similar to its effect on type I IFN, miR-21 suppressed the HCV-induced production of interleukin (IL)-6, tumor necrosis factor (TNF)-α, and IL-8 (Figure S3D and S3E). Moreover, miR-21 decreased HCV-induced NF-κB p65 phosphorylation, indicating that NF-κB signaling is also negatively regulated by miR-21 (Figure S3F). These results suggest that miR-21 downregulates a range of responses to HCV infection in hepatocytes.

### miR-21 facilitates HCV replication by counteracting antiviral activity of IFN-α

**Figure 6:**
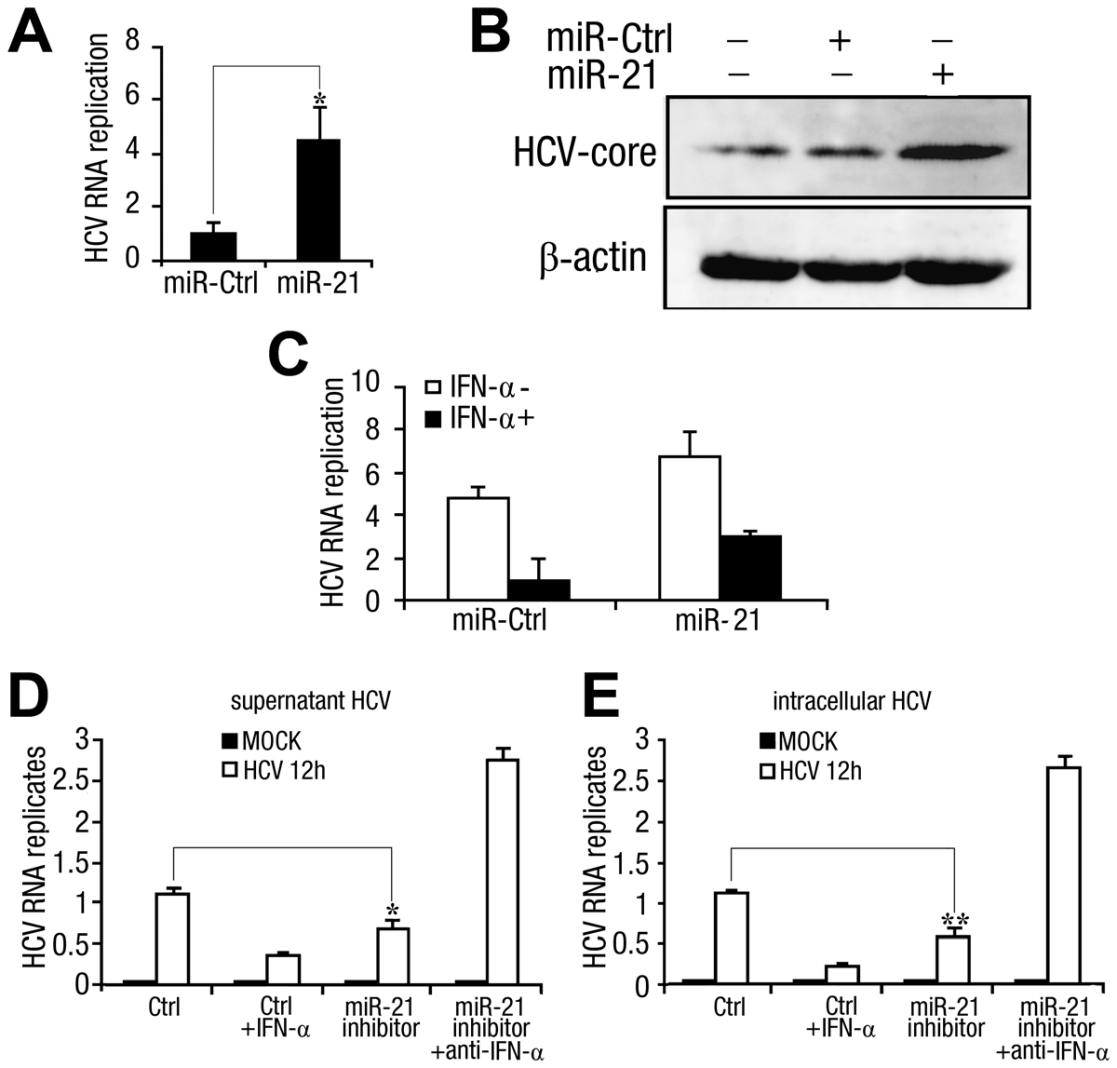
miR-21 stimulates HCV replication and attenuates the HCV response to IFN-α treatment. (*A*) Huh7 hepatocytes were transfected with control RNA (miR-ctrl) or miR-21 mimics (final concentration, 50 nM). After 48 h, cells were infected with HCV (MOI = 1) for 2 h and washed before fresh medium was added. After 72 h, intracellular HCV RNA replicates were quantified by qPCR and normalized to the GAPDH internal control. (*B*) Huh7 hepatocytes were transfected as described in (*A*) and infected with HCV (MOI = 1) for 2 h. After 48 h, HCV core expression was analyzed by Western blot (top panel) using β-actin as a loading control (bottom panel). (*C*) Huh7 hepatocytes were transfected with miR-21 mimics or control RNA (miR-ctrl) (final concentration, 50 nM). After 48 h, cells were infected with HCV (MOI = 1) for 2 h and washed before adding fresh medium with or without recombinant human IFN-α (100 U/ml). After 72 h, intracellular HCV RNA replicates were quantified by qPCR. (*D and E*) Huh7 hepatocytes were transfected with miR-21 inhibitors or control inhibitor (ctrl) (final concentration, 50 nM). After 48 h, cells were infected with HCV (MOI = 1) for 2 h and washed before adding fresh medium with or without recombinant human IFN-α (100 U/ml) or anti-IFN-α-neutralizing antibody (100 neutralizing units/ml) as indicated. After 72 h, RNA was isolated from the cell culture medium, and supernatant HCV replicates (*D*) were measured by qPCR. Intracellular HCV RNA replicates (*E*) were quantified by qPCR using GAPDH as internal control. Data are presented as the means
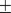
SD (n = 3) from one representative experiment. Similar results were obtained in three independent experiments. **, p<0.01; *, p<0.05.

To determine whether miR-21 facilitates HCV replication and expression, Huh7 cells were transfected with miR-21 and then infected with HCV (MOI = 1). Intracellular HCV RNA levels were determined by qPCR, and HCV core protein expression was determined by Western blot analysis. The results showed that miR-21 overexpression significantly upregulated HCV replication and core protein expression (Figure 6A and B), indicating that miR-21 can facilitate HCV replication.

To evaluate the effects of IFN-α on HCV replication in the presence of miR-21, Huh7 cells were transfected with either miR-21 or miR-Ctrl and then infected with HCV, followed by treatment with or without IFN-α (100 U/ml). The results from qPCR analysis showed that there was a 5-fold reduction in HCV replication after IFN-α treatment in control cells, but only a 2-fold reduction in HCV replication of miR-21-overexpressing cells (Figure 6C). These results demonstrate that miR-21 counteracts the effects of IFN-α treatment on viral replication.

To further characterize the biological significance of the observed up-regulated miR-21 expression and the subsequent impairment of HCV-triggered type I IFN production, we further investigated the effects of inhibiting the miR-21-inducible expression on HCV replication and the role of IFN-α. We transfected HCV-infected Huh7 cells with the miR-21 inhibitor and determined the level of HCV replication in the cell culture medium by qPCR. We found that inhibiting miR-21-inducible expression, the same as by adding of recombinant IFN-α, suppressed HCV replication; however, this effect was reversed by anti-IFN-α-neutralizing antibodies, suggesting that miR-21 promotes HCV replication by downregulating IFN-α production (Figure 6D). Intracellular HCV replicates were similarly affected by the miR-21 inhibitor and anti-IFN-α-neutralizing antibodies (Figure 6E). These results demonstrate that HCV-induced miR-21 expression promotes HCV replication in hepatocytes by suppressing type I IFN production.

### The antiviral responsiveness of IFN-α is attenuated by miR-21 overexpression

**Figure 7:**
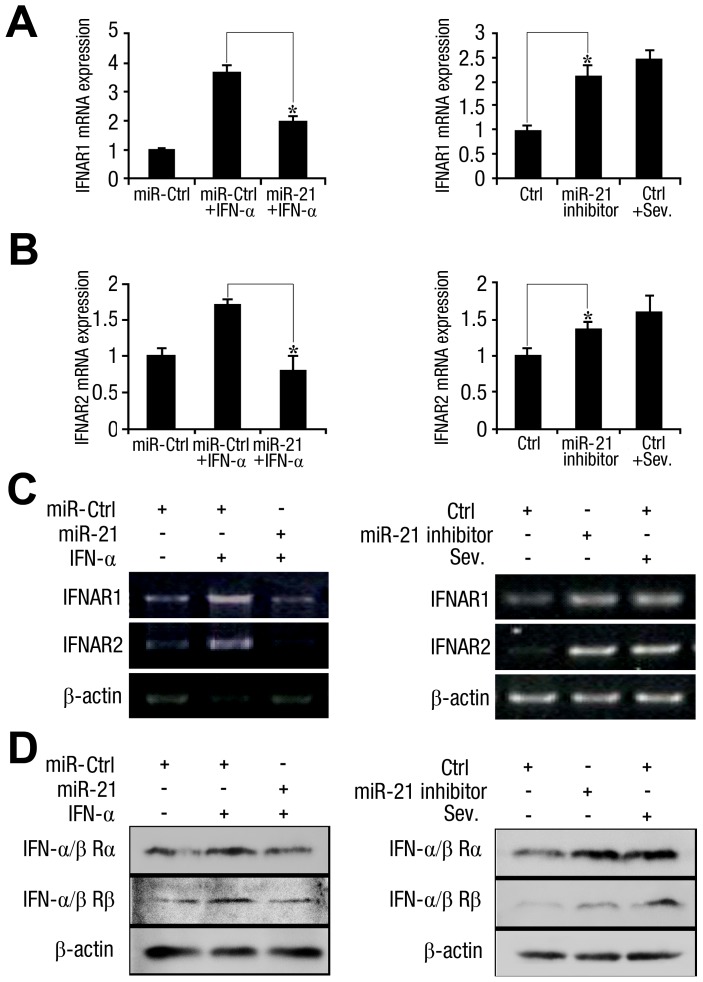
The antiviral IFN-α response is downregulated by miR-21 in hepatocytes. Huh7 hepatocytes were transfected with miR-21 mimics or control mimics, miR-21 inhibitor or control inhibitor (final concentration, 50 nM), as indicated. After transfection for 30 h, the cells were treated with recombinant human IFN-α (100 U/ml) or infected with Sendai virus (SeV), as indicated. After 12 h, IFNAR1 and IFNAR2 mRNA and protein expression was determined by qPCR (*A*, *B*), RT-PCR (*C*) and Western blot analysis (*D*), respectively. Data are shown as the means
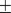
SD (n = 3) from one representative experiment. Similar results were obtained in three independent experiments. **, p<0.01; *, p<0.05.

The IFN-α/β subtypes activate a common type I IFN receptor, which consists of two subunits (IFNAR1 and IFNAR2). The type I IFN receptor is expressed on the surface of virtually all host cells [33] and is essential for the antiviral activity of IFN-α [34], [35]. We used qPCR, RT-PCR, and Western blot analyses to elucidate the effects of miR-21 on IFNAR1/IFNAR2 expression. The Sendai virus (SeV), which triggers the IFN response, was used as a positive control. As shown in Figure 7, IFNAR1/IFNAR2 expression was significantly reduced in cells overexpressing miR-21 (left/top panel) and increased in cells expressing the miR-21 inhibitor (right/low panel).

Binding of type I IFN to IFNAR initiates a signaling cascade that leads to the induction of more than 300 IFN-stimulated genes [36]. Signal transducer and activator of transcription (STAT) proteins are transcription factors that are important in IFN signaling; genetic defects in STAT-1 result in death by viral disease at an early age [35]. Double-stranded RNA-activated protein kinase (PKR), oligoadenylate synthetase (OAS), and myxovirus resistance protein (Mx) also mediate the antiviral effects of IFN-α [37]–[39]. Therefore, we assessed the effect of miR-21 on these proteins in Huh7 cells. Our results demonstrated that miR-21 attenuated the IFN-induced phosphorylation of STAT1/STAT2 (Figure S4A) and the expression of PKR, OAS, and Mx (Figure S4B). In addition, we observed that although the phosphorylation of STAT1 and STAT2 was downregulated by miR-21, the total STAT protein levels were relatively unaffected, indicating that JAK-STAT signaling activity is negatively regulated by miR-21. These results demonstrate that miR-21 suppresses the antiviral response mediated by IFN-α.

### miR-21 regulates the expression of components of the Toll-like receptor signaling cascade

**Figure 8:**
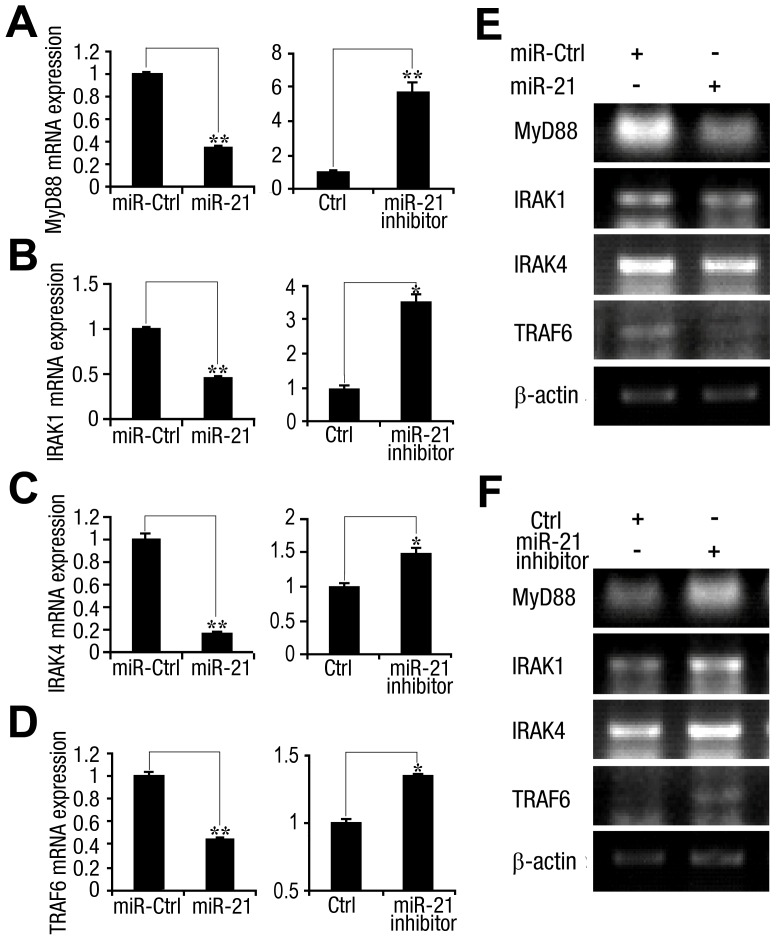
miR-21 regulates components of the Toll-like receptor 7 signaling cascade. Huh7 hepatocytes were transfected with miR-21 mimics or control RNA, miR-21 inhibitor or control inhibitor (final concentration, 50 nM). After 48 h, MyD88, IRAK1, IRAK4, and TRAF6 mRNA levels were determined by qPCR (*A*, *B*, *C*, and *D*) and RT-PCR (*E* and *F*), respectively. Data are presented as the means
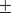
SD (n = 3) from one representative experiment. Similar results were obtained in three independent experiments. **, p<0.01; *, p<0.05.

After detection of viral ssRNA, the endosomal single-stranded RNA receptor, Toll-like receptor 7 (TLR7), uses the MyD88 adaptor protein, to initiate IFN-α synthesis *via* interleukin-1-receptor-associated kinase (IRAK) 1, IRAK4, tumor necrosis factor receptor-associated factor (TRAF6), and the interferon regulatory factor (IRF) 7 transcription factor [40]. TLR7 is expressed in HCV-infected hepatocytes [41], therefore, we evaluated proteins in this signaling cascade to identify potential targets of miR-21. As shown in Figure 8, the mRNA levels of MyD88, IRAK1, IRAK4, and TRAF6 were decreased by miR-21 overexpression and increased by miR-21 inhibition. We also evaluated the gene expression of TLR7 in the same experimental samples using qPCR and reverse transcription (RT)-PCR. TLR7 expression did not show any significant changes in hepatocytes in which miR-21 was over-expressed or silenced compared with the control cells transfected with miRNA-control (data not shown). Since miRNA mimics were small chemically modified double-stranded RNAs, and the sequence of miR-21 mimic did not contain the 5′-UGUGU-3′ internal motif [42], we deduced that miR-21 mimic could not activate TLR7 signaling as ssRNA.

IRF-7 is an ISG that is expressed in HCV-infected hepatocytes, and its expression in the liver is IFN-dependent [43], [44]. IRF-7 promotes the expression of various IFN subtypes, which further amplifies the host response and prolongs IFN production [44]. Here, we investigated the effect of miR-21 on the expression of IRF-7. qPCR (Figure S5A) and RT-PCR (Figure S5B) results showed that the levels of IRF-7 mRNA were decreased upon treatment with miR-21 and increased upon treatment with miR-21 inhibitor. The results of a Western blot analysis showed that the nuclear translocation of IRF7 was attenuated by miR-21 (Figure S5C, left panel) and stimulated by miR-21 inhibitor (Figure S5C, right panel). These results suggested that miR-21 mediates host antiviral responses by suppressing the expression of genes involved in the TLR signaling cascade.

Additionally, as TLR signaling components were found to be regulated by HCV-induction of miR-21, HCV infection itself may also reduce the expression of these signaling components. Following treatment of Huh7 cells with FL-J6/JFH5′C19Rluc2AUbi for 24 h, qPCR results confirmed our hypothesis (Figure S6A). The same effect was shown with respect to the IRF7 nuclear translocation mediated by HCV activation (Figure S6B).

### MyD88 and IRAK1 are targets of miR-21

**Figure 9:**
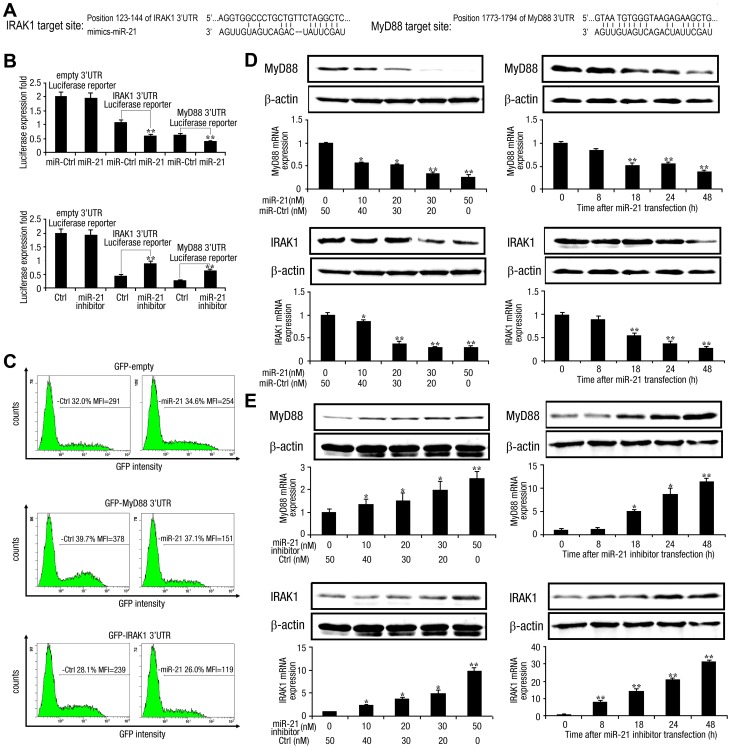
miR-21 targets human MyD88 and IRAK1. (*A*) Sequence alignment of miR-21 and its binding sites in the 3′ UTRs of MyD88 and IRAK1, as predicted by RNA22 software. (*B*) Huh7 hepatocytes (1×10^4^) were co-transfected with pGL3-Basic, pGL3-MyD88 3′ UTR, or pGL3-IRAK1 3′ UTR firefly luciferase reporter plasmids (80 ng) and pRL-TK *Renilla* luciferase plasmid (40 ng), together with miR-21 mimics or control RNA, miR-21 inhibitor or control inhibitor (final concentration, 50 nM), as indicated. After 48 h, firefly luciferase activity was determined and normalized to *Renilla* luciferase activity. (*C*) HEK293 cells (1×10^4^) were co-transfected with GFP control, GFP-MyD88 3′ UTR, or GFP-IRAK1 3′ UTR plasmid (400 ng), together with miR-21 mimics or control RNA (final concentration, 50 nM), as indicated. After 48 h, GFP expression was analyzed by FACS, and the mean fluorescence intensity (MFI) of GFP was determined. (*D* and *E*) Huh7 hepatocytes (1×10^6^) were transfected with miR-21 mimics (*D*) or miR-21 inhibitor (*E*) at various concentrations for 48 h (*left*), or at 50 nM (final concentration) for the indicated time (*right*). MyD88 and IRAK1 protein levels were determined by Western blot and normalized to β-actin (*top panel*); MyD88 and IRAK1 mRNA levels were determined by qPCR and normalized to GAPDH (*bottom panel*). Data are presented as the means
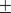
SD (n = 3) from one representative experiment. Similar results were obtained in three independent experiments. **, p<0.01; *, p<0.05.

Next, we investigated potential targets of miR-21 involved in modulating HCV-triggered type I IFN production. We used RNA22 and FINDTAR3 software to predict miR-21 targets and found putative miR-21 binding sites in the 3′ UTRs of human MyD88 and IRAK1 (Figure 9A). To verify the possibility that MyD88 and IRAK1 were regulated post-transcriptionally by miR-21, we constructed reporter plasmids by cloning these miR-21 binding sites into the 3′ UTRs of firefly luciferase or green fluorescent protein (GFP). A four base pair mutation (mutant type) was introduced into the miR-21 binding site within the 3′UTR of IRAK1 or MyD88 cDNA (Figure S7A). After co-transfecting the luciferase reporter plasmids into Huh7 cells with vectors expressing miR-21 mimics or inhibitors, we observed that miR-21 mimics markedly decreased luciferase expression, whereas miR-21 inhibitors increased luciferase expression (Figure 9B). miR-21 mimics also downregulated GFP gene expression when the 3′-UTR of MyD88 or IRAK1 was cloned into the 3′-UTR region of GFP in HEK293 cells, as assessed by flow cytometry (Figure 9C). Paralleling the previous results, neither luciferase nor GFP gene expression could be regulated by miR-21 with a mutant-type 3′UTR (Figure S7B and S7C). Furthermore, transfection of miR-21 mimics decreased MyD88 and IRAK1 expression in Huh7 cells in a dose-dependent and time-dependent manner (Figure 9D), whereas miR-21 inhibitors increased MyD88 and IRAK1 expression (Figure 9E), suggesting that MyD88 and IRAK1 expression could be inhibited by miR-21 via both translational inhibition and mRNA degradation. Taken together, these results show that miR-21 regulates the expression of MyD88 and IRAK1, suggesting the possibility that human MyD88 and IRAK1 are new targets of miR-21.

Besides, we confirmed that HCV alone does, indeed, downregulate MyD88 and IRAK1 expression (Figure S6C). The suppression of MyD88 and IRAK1 caused by HCV infection was almost eliminated in miR-21 inhibitor transfected cells but not in negative inhibitor control transfected cells or the mock transfected cells at 24 h.p.i. (Figure S6D). This result was in agreement with our hypothesis that upregulation of miR-21 by HCV results in the downregulation of these two factors.

### Regulation of IFN signaling by miR-21 is mediated by MyD88 and IRAK1

**Figure 10:**
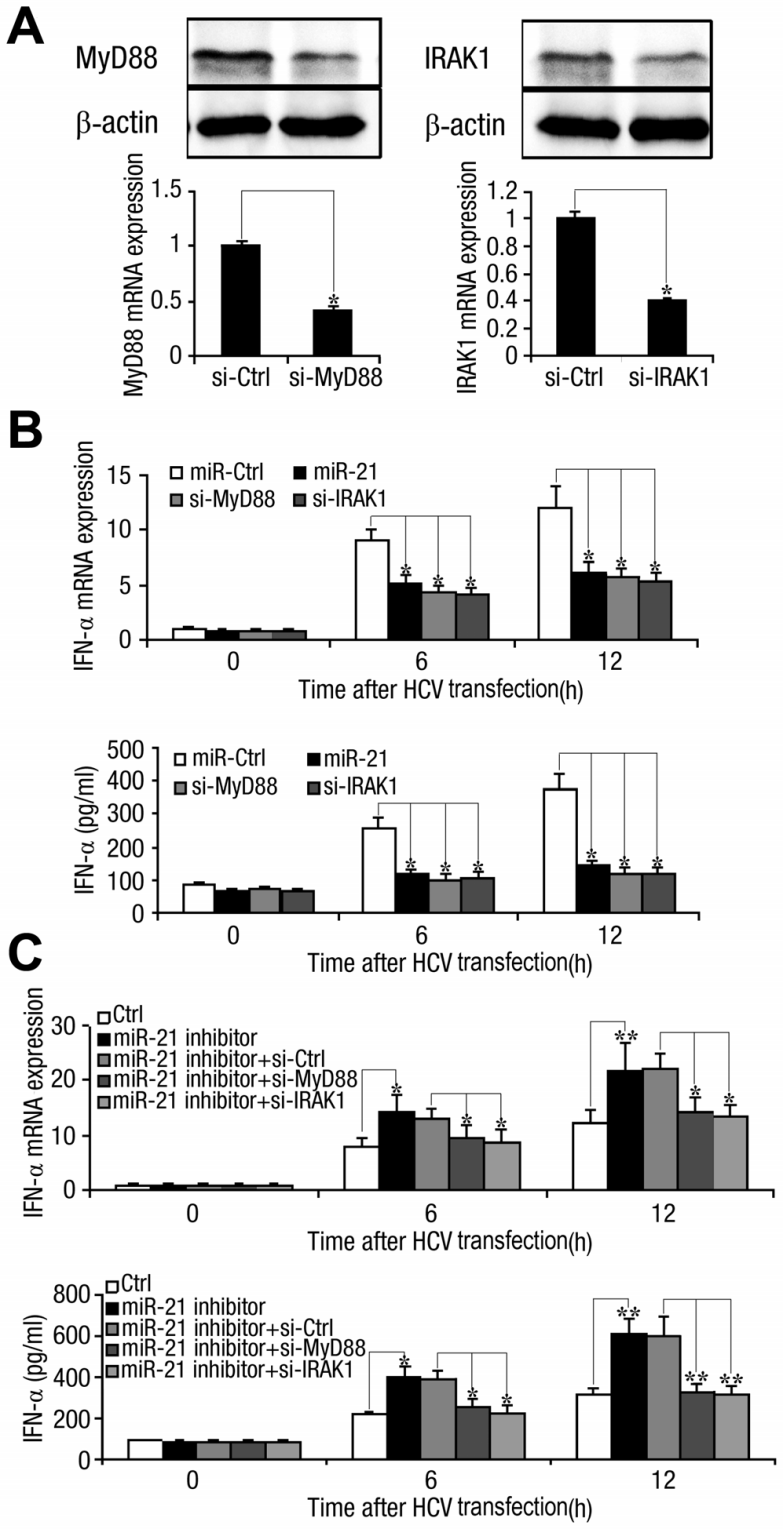
Regulation of IFN signaling by miR-21 is achieved primarily through MyD88 and IRAK1. (*A*) Huh7 hepatocytes were transfected with nonspecific control siRNA or siRNA against MyD88 or IRAK1, as indicated. After 24 h, MyD88 and IRAK1 mRNA levels were determined by qPCR and normalized to GAPDH (*lower panel*); after 48 h, MyD88 and IRAK1 protein levels were determined by Western blot and normalized to β-actin (*upper panel*). (*B*) Huh7 hepatocytes were co-transfected with miR-21 mimic or control RNA and siRNA against MyD88 or IRAK1, as indicated. After 48 h, Huh7 cells were transfected with FL-J6/JFH5′C19Rluc2AUbi (0.1 µg) for the indicated time, and IFN-α expression and secretion were determined by qPCR and ELISA, respectively. (*C*) Huh7 hepatocytes were co-transfected with miR-21 inhibitor or control inhibitor and siRNA against MyD88 or IRAK1 or nonspecific control siRNA, as indicated. After 48 h, Huh7 cells were transfected with FL-J6/JFH5′C19Rluc2AUbi (0.1 µg) for the indicated times, and IFN-α expression and secretion were determined by qPCR and ELISA, respectively. Data are the means
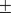
SD (n = 3) of one representative experiment. Similar results were obtained in three independent experiments. **, p<0.01; *, p<0.05.

To verify the roles of MyD88 and IRAK1 in IFN signaling, we silenced these genes and examined the effects on HCV-triggered type I IFN production. Small interfering RNAs (siRNAs) effectively inhibited MyD88 and IRAK1 expression in Huh7 cells (Figure 10A). Knockdown of MyD88 or IRAK1 significantly decreased the levels of IFN-α mRNA and protein (Figure 10B), which produced effects similar to those of miR-21 overexpression. Increased IFN-α expression due to miR-21 inhibition was suppressed by siRNAs against MyD88 or IRAK1 (Figure 10C). These results suggest that knockdown of MyD88 or IRAK1 phenocopied the attenuated IFN signaling effect of induced miR-21 and counteracted the effect of miR-21 inhibition. Taken together, these results indicate that virally induced miR-21 regulates IFN signaling through suppression of endogenous MyD88 and IRAK1, thereby inhibiting type I IFN production.

It is known that type I IFN production through the Toll-like-interleukin-1 receptor domain-containing adaptor (TRIF) plays a crucial role in mounting an antiviral response against HCV and the virus has evolved to cripple this pathway [45]. To test whether this pathway also affect the IFN-α meditated by the miR-21 on MyD88 pathway, TRIF or IRF was knocked down in Huh7 cell lines, which were then treated with miR-21 and HCV. The level of IFN-α protein was decreased by miR-21 and this regulation was not affected by treatment with TRIF or IRF3 siRNA compared to the control (Figure S8). Thus, the mechanism by which miR-21 uses MyD88 and IRAK1 to counter the type I IFN-associated antiviral response during HCV infection is an independent pathway of the TRIF pathway.

### miR-21 enhances the replication and production of other viruses by hampering the antiviral activity of IFN-α

Because miR-21 disrupts antiviral function by inhibiting type I IFN, we speculated that miR-21 may exhibit a broad range of functions involved in regulating the replication and production of other viruses. To confirm this hypothesis, three additional viruses, EV71, HIV-1, and VSV, were included in our analyses.

**Figure 11:**
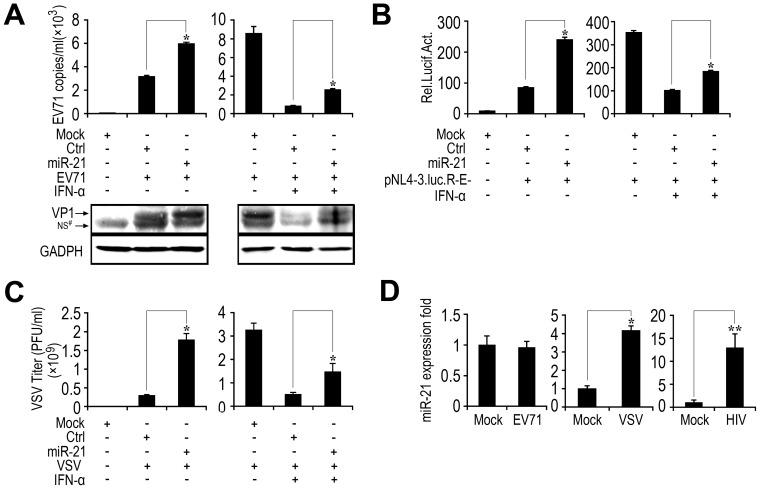
Effects of other viruses on the regulation of miR-21 expression. (*A*) RD cells were transfected with miR-21 mimics or control RNA (final concentration, 50 nM) and further treated with or without IFN-α (100 U/ml) 24 h later. Thirty-six hours after transfection, the cells were infected with EV71 (MOI = 1) for 2 h and then washed. Cells were harvested at 12 h post-infection. EV71 RNA levels were quantified by qPCR (*upper panel*), and the viral VP1 protein expression was measured by Western blot (*lower panel*). (*B*) PBMCs were co-transfected with pNL4-3.luc.R-E- and miR-21 mimics or control RNA (final concentration, 50 nM) for 24 h and treated with or without IFN-α (100 U/ml). Luciferase activity was then measured. (*C*) Huh7 cells were transfected with miRNA or control RNA as indicated (final concentration, 50 nM). After 24 h, cells were infected with VSV (MOI = 1) for 2 h and washed before treatment with or without IFN-α (100 U/ml). Supernatants were harvested at 24 h post-infection and analyzed for VSV production using standard plaque assays. (*D*) VSV and HIV, but not EV71 induced miR-21 expression. RD cells were infected with EV71 (MOI = 1) for 6 h. Huh7 cells were infected with VSV (MOI = 1) for 6 h. PBMCs were transfected with pNL-luc-E-R+ plasmids for 48 h. miR-21 expression was determined by qPCR. Experiments were performed three times with similar results. All graphs represent means ± SD, n = 3. **, p<0.01; *, p<0.05.

First, we examined the effects of miR-21 on EV71 replication in RD cells. miR-21 effectively stimulated viral VP1 mRNA and protein levels (Figure 11A left panel). The suppression effect of IFN-α treatment on virus replication was weakened by miR-21 over-expression (Figure 11A, right panel). Next, we assessed the effects of miR-21 on HIV production in peripheral blood mononuclear cells (PBMCs). A reporter plasmid containing an HIV-1 clone, pNL4-3.luc.R-E-, was introduced into PBMCs together with a control Renilla luciferase-expressing plasmid (pRL) as a loading control. Luciferase activity was measured in the presence of cotransfected synthetic miR-21 or negative control, treated with or without IFN, respectively. The results from the luciferase assays showed that HIV-1 transcription was induced in the presence of miR-21(Figure 11B, left panel), and miR-21 also counteracted the IFN-α anti-HIV activity (Figure 11B, right panel). VSV is a pathogen that is extremely sensitive to the action of type-I IFNs. Thus, we finally investigated the effect of miR-21 on VSV production. Similar to the results obtained for EV71 and HIV, miR-21 overexpression in Huh7 cells resulted in increased virus titers (Figure 11C, left panel), and decreased the effects of IFN-α on VSV.

These results suggested that miR-21 activates the replication of EV71, HIV-1, and VSV by suppressing the antiviral activity of IFN-α. The effects of EV71, HIV-1, and VSV on the expression of miR-21 were also investigated in this study. RD, PBMCs, and Huh7 cells were infected with EV71, HIV-1, or VSV, respectively. qPCR results indicated that the levels of miR-21 were induced significantly by both HIV-1 and VSV, but not by EV71.

## Discussion

In response to viral infection, signaling pathways in mammalian cells direct a variety of intracellular events that generate an immune response within infected cells. This host response represents our first line of defense against viral infection, and if successful, results in an abortive infection. However, viruses adopt various strategies to evade host defenses [46]. Molecular mechanisms by which viruses trigger and regulate these antiviral processes have recently been characterized. The host response launches an innate immune defense against HCV infection [47], and HCV evades the host response through a multifaceted process that includes signaling interference, effector modulation, and continuous genetic variation. These evasion strategies promote the persistent infection and spread of HCV; therefore, identifying the precise molecular mechanisms by which HCV regulates the host response may reveal novel therapeutic targets. The TLR-MyD88 signaling pathway plays pivotal roles in virus recognition and initiation of the antiviral response in immune cells. Although HCV was previously shown to modulate the TLR-MyD88 antiviral response [48], the precise mechanism involved remains unclear, and additional negative regulators need to be identified.

miR-21 is overexpressed in breast, colon, lung, pancreas, prostate, and stomach tumors, as well as in cholangiocarcinoma cell lines, indicating that miR-21 may play a key role in tumor cell behavior and malignant transformation. Inhibition of miR-21 also increases cell growth in other cancer types [49] and exerts anti-apoptotic effects in glioblastoma and cholangiocarcinoma [50], [51]. Thus, altering miR-21 expression can produce diverse effects on tumor cells. Here, we revealed that miR-21 expression is stimulated by HCV infection in human hepatocytes, and showed that miR-21 is also activated in cells infected HIV-1 or VSV. Thus, we provided a direct evidence for the association of miR-21 expression with viral infection.

The mechanism involved in the activation of miR-21 mediated by HCV infection was further investigated. Our results showed that AP-1 is at least partially responsible for the upregulation of miR-21. Recently, some researchers have demonstrated that AP-1 activates the miR-21 transcription in conjunction with the switch/sucrose non fermentable (SWI/SNF) complex, after phorbol 12-myristate 13-acetate (PMA) stimulation through the conserved AP-1 and PU.1 binding sites in the promoter. Hence, it is possible that posttranscriptional processing and/or decay of miRNAs may also contribute to the unequal upregulation of miR-21 during HCV infection. AP-1 is a cellular transcription factor complex involved in several cellular functions, such as cell proliferation, apoptosis, and differentiation. It has recently been reported that AP-1 is induced during infections by various viruses, including hepatitis B virus, Epstein-Barr virus (EBV), herpes simplex virus type 1 (HSV-1), and HIV, and that this induction promotes viral replication. Many viral proteins can induce multiple signaling pathways, which may cross-talk with each other or converge on common downstream effectors. In the present study, we demonstrated that AP-1 is essential for NS5A and NS3/4A protein-induced miR-21 expression, as mutations in AP-1 binding sites eliminated the protein-induced activation of miR-21 promoter activity. Based on our finding that the NS5A and NS3/4A proteins activate c-Jun and c-Fos expression, respectively, we further investigated the roles of different MAP kinases in the activation of miR-21 by the two proteins. The results suggested that ERK, JNK, and PKC were all involved in the HCV-mediated activation of miR-21. Additionally, active PKCα/ERK was required for the binding of c-Fos to the miR-21 promoter in response to NS3/4A protein stimulation; the PKCε/JNK pathway is the important signaling pathway in the regulation of c-Jun by the NS5A protein. According to our confocal immunofluorescence results, NS3/4A and NS5A were distributed in the cytoplasm evenly (Figure S10), and this observation was consistent with the previous results [52], [53]. However, there is also evidence that the nuclear localization signal (NLS) of NS5A can be detected in the nucleus, though the full-length NS5A protein is localized in cytoplasm [54]. This, therefore, suggests that NS5A may have the ability to translocate into the nucleus and function as an activator of transcription when the NLS is exposed.

Several genes have been identified as miR-21 targets, suggesting the multifaceted function of miR-21; however, the target profile of miR-21 does not appear to be complete. The major targets and functions of a specific miRNA vary under different physiological or pathological conditions and in different cell types. miR-21 appears to participate in various biological processes by targeting different genes, including PTEN [24], DNMT1 [55], PDCD4 [56] and FasL [57]. The abundance of different miRNAs following HCV infection has already been examined [58], [59], and the overexpression of miR-21 has also been shown. However, the upregulation of miR-21 to downregulate specific proteins involved in the IFN response has not been described. In the present study, we demonstrated that miR-21 functions as a negative regulator, targeting MyD88 and IRAK1 in HCV-infected hepatocytes to attenuate IFN signaling, thereby facilitating HCV evasion of antiviral responses. Our findings contribute to the understanding of the role of miR-21 in host–virus interactions.

We found that miR-21 attenuated the expression of the IFN-stimulated genes PKR, Mx, and OAS (Figure S4B), which limit HCV replication by disrupting viral RNA translation and inhibiting viral genomic RNA synthesis [60]–[62]. We then checked upstream by examining the phosphorylation status of STAT1 and STAT2. miR-21 decreased STAT1/STAT2 phosphorylation, but had little effect on total STAT protein levels (Figure S4A). The activation of STAT1 and STAT2 mainly occurs through the IFN-JAK/STAT pathway, so we evaluated the initiation point of this pathway, IFN-associated receptors (IFNARs), and found that miR-21 overexpression reduced IFNARs expression (Figure 7). According to these results, the induction of miR-21 can disturb the JAK-STAT pathway, which is a logical reason for its upregulation on HCV upon replication.

Activation of certain TLRs induces type I IFNs, which are known to inhibit HCV infection. Upon binding with a specific ligand, most TLRs recruit the common TLR adaptor MyD88, which in turn recruits IRAK and TRAF6 proteins and triggers MAPK and NF-κB signaling to produce pro-inflammatory cytokines [63], [64]. Therefore, suppression of MyD88 and IRAK1 by inducible miR-21 attenuates antiviral immunity and renders the immune response less effective.

Several viral proteins also counteract TLRs and their downstream signaling events. Measles virus and respiratory syncytial virus inhibit TLR7-dependent and TLR9-dependent IFN signaling stimulated by resiquimod and CpG oligodeoxynucleotides in primary human plasmacytoid dendritic cells [65]. Here, we report the novel regulation of TLR-MyD88 signaling by miRNA. First, we found that HCV infection upregulated miR-21 expression in hepatocytes. Second, we demonstrated that miR-21 suppresses HCV-triggered type I IFN production, thereby promoting HCV replication, and thus, demonstrating a novel mechanism for viral immune evasion. Third, we demonstrated that both human MyD88 and IRAK1 are targets of miR-21. In this new model of HCV infection, HCV is first identified by TLRs and retinoic-acid-inducible gene-1 (RIG-1), which in turn initiate type I IFN production against HCV infection. Meanwhile, HCV infections can also upregulated the expression of miR-21, which counteracted the IFN-mediated antiviral response by inhibiting TLR-MyD88 signaling. Consistently, miR-21 overexpression stimulated replication and production of HCV, VSV, HIV and EV71. Intriguingly, unlike the other three viruses, levels of miR-21 were not induced significantly by EV71 (Figure 11). As it has been reported that EV71 fails to stimulate the expression of type I IFN in mammalian cells [66], the upregulation of miR-21 expression may not be required after EV71 infection. Induction of miR-21 expression may be a new survival strategy of the virus to escape the antiviral immune response of the host.

The host response is the first line of immune defense against viral pathogens, and it imposes several barriers that HCV must subdue to replicate and persist. HCV evades the host response through a complex combination of virus-host interactions that disrupt intracellular signaling pathways and attenuate the antiviral actions of IFN. Viral regulation of the host response impairs the crosstalk between innate and adaptive immunity and provides a foundation for HCV replication and spreading. It is generally thought that miRNAs target multiple mRNAs, termed the targetome, to regulate gene expression. A single miRNA might fine-tune the protein synthesis of thousands of genes directly or indirectly [67]. In the present study, human MyD88 and IRAK1 were shown to be novel targets of miR-21, contributing to the negative regulatory feedback function of miR-21 in TLR-MyD88 signaling. Taken together, our results suggest that miR-21 may be a potential therapeutic target for antiviral intervention; however, this needs to be verified in future studies.

The interaction between the HCV and TLR/RIG-I pathways is well documented, and expression of the HCV NS5A and NS3/4A proteins may disrupt TLR-dependent and TLR-independent signaling pathways, respectively. For example, the NS3/4A protease disrupts the TLR3 and RIG-I cascades by cleaving the essential adaptor proteins TRIF and mitochondrial antiviral signaling protein (MAVS), respectively [45], [68]. Furthermore, the NS5A protein was shown to inhibit the TLR-MyD88 signaling pathway through a direct interaction with the death domain of MyD88 through the interferon sensitivity-determining region (ISDR) [48]. In this study, we found that the functional mechanism of miR-21 action through MyD88 and IRAK1, which was also modulated by NS3/4A and NS5A protein expression, could be another mechanism independent pathway of the TRIF pathway for impairing the type I IFN-associated antiviral response during HCV infection (Figure S7).

**Figure 12:**
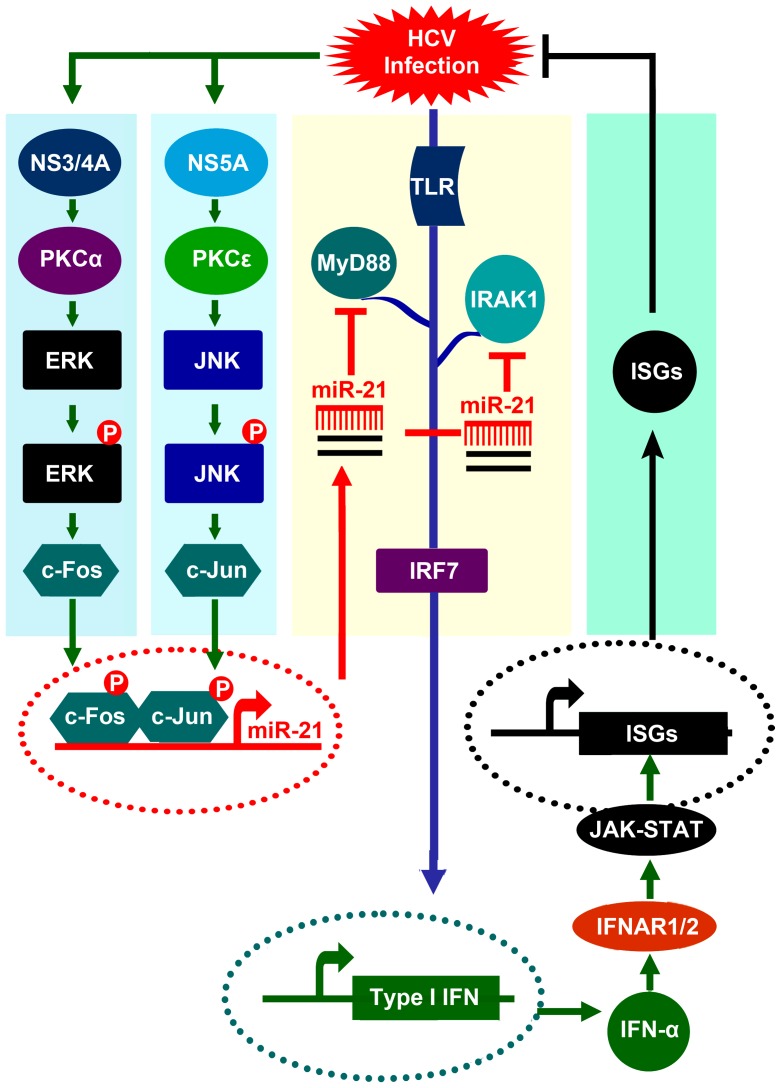
Proposed model for the activation of miR-21 expression upon HCV infection and the role of miR-21 in the regulation of the antiviral activity of type I IFN. During HCV infection, the virus is first recognized by TLRs and RIG-1, which in turn activates MyD88 and IRAK1 to initiate IFN-α synthesis, resulting in the activation of ISGs and the inhibition of HCV replication. In addition, miR-21 expression is activated during HCV infection through two signaling pathways: the PKCε/JNK/c-Jun pathway and the PKCα/ERK/c-Fos pathway. The HCV NS5A protein activates PKCε to enhance the expression of JNK and c-Jun, while the HCV NS3/4A complex stimulates PKCα to promote the production of ERK and c-Fos. The two subunits (c-Jun and c-Fos) of AP-1 join together to recognize the miR-21 promoter and activate the expression of miR-21, which represses the expression of MyD88 and IRAK1 via imperfect base pairing between miR-21 and the 3′UTR of MyD88 and IRAK1. The reduction in MyD88 and IRAK1 causes a reduction of type-I IFN production and ISG expression that might contribute to viral pathogenesis and virus propagation.

In conclusion, we have identified a virus-host interaction pathway wherein HCV infection results in the stimulation of two signaling pathways: the NS5A/PKCε/JNK/c-Jun pathway and the NS3/4A/PKCα/ERK/c-Fos pathway. After infection, c-Jun and c-Fos bind to the AP-1 binding sites in the miR-21 promoter and evoke AP-1 mediated miR-21 induction, leading to the silencing of MyD88 and IRAK1, which play a major role in the initiation of the antiviral response in host cells and increased virus production (Figure 12).

## Materials and Methods

### Ethics statement

All research involving human participants was approved by the Institutional Review Board of the College of Life Sciences, Wuhan University, China, in accordance with their guidelines for the protection of human subjects. Written informed consent was obtained from each participant.

### Reagents

Antibodies against HCV-core, STAT1, IRF-7, PKR, OAS, Mx, IFN-α/β Rα, IFN-α/β Rβ, phosphor-NF-κB p65, NF-κB p65, phosphor-ERK, ERK, phosphor-JNK, JNK, phosphor-c-Fos, c-Fos, phosphor-c-Jun, c-Jun, MyD88, IRAK1, and anti-mouse normal IgG were purchased from Santa Cruz Biotechnology (Santa Cruz, CA, USA). Antibodies against phospho-STAT1, STAT2, and phospho-STAT2 were purchased from Cell Signaling Technology (Cell Signaling Technology, Beverly, MA, USA). The actin antibody was purchased from CWBio (CWBIO, Beijing, China), and the human IFN-α antibody and IFN-α-neutralizing antibody were purchased from PBL Interferon Source (PBL, Piscataway, NJ).

The following inhibitors used in this study were used: LY-294002, H-89, Sp600125, U0126, PD98059, SB203580, MG132 and GF10203 were purchased form Tocris Bioscience (Tocris Bioscience, Missouri, USA). ALL of the inhibitors were dissolved in dimethylsulfoxide (DMSO; Sigma, Abitibi belt, Canada).

Sendai virus was a gift from Dr. Shu Hong-Bing (Wuhan University, China). HCV genotype 2a (JFH-1) strain was kindly provided by Dr. Takaji Wakita (Tokyo Metropolitan Institute for Neuroscience, Japan). HCV replicon plasmid FL-J6/JFH-5′C19Rluc2AUbi, which carries a monocistronic full-length viral genome that expresses Renilla luciferase (Rluc) was reported previously [69]. This plasmid was a kind gift from Charles M. Rice (Rockefeller University, USA). The Indiana serotypes of VSV were provided by the China Center Type Culture Collection. Enterovirus 71 (EV71) was provided by the Renmin Hospital of Wuhan (Hubei, China). The HIV-1 clone NL4-3.Luc.R-E- competent for a single round of replication, which is vpr- and env-, with nef substituted by luciferase (He 995), was obtained through the National Institute of Health (NIH) AIDS Research and Reference Reagent Program. pNL4-3/Udel with vpu deletion was kindly provided by Dr. Klaus Strebel (National Institute of Allergy and Infectious Disease, NIH). Mutants of ERK1 and ERK2 were gifts from Dr. Cobb (University of Texas Southwestern Medical Center) and mutants of JNK were gifts from Dr. Karin (University of California at San Diego, San Diego, CA, USA). The PKC RNAi vectors were constructed by ligating the corresponding pairs of oligonucleotides to pSilencer 2.0 (Ambion, Inc., Austin, TX, USA) based on the sequences described by Storz *et al.* Plasmids expressing HCV genotype 2a proteins were generated in the State Key Laboratory of Virology, Wuhan University, as described previously [70].

### Cell culture and transfection

Huh7 human hepatoma and HEK293 human embryonic kidney cells were grown in Dulbecco's Modified Eagle's Medium (DMEM) supplemented with 10% heat-inactivated fetal calf serum, 100 U/ml penicillin, and 100 µg/ml streptomycin sulfate at 37°C in 5% CO_2_. Cells were plated in 6-well plates (4.0×10^5^ cells/well) and grown to 80% confluence. Then, the cells were seeded into 24-well plates (2×10^5^ cells/well) and incubated overnight. Cells were subsequently transfected with Lipofectamine (Invitrogen) according to the manufacturer's instructions. Levels of expression of transfected HCV proteins were similar to those of endogenous HCV proteins during peak viral expression (Figure S9). Transfection with synthetic oligonucleotides did not exhibit significant cytotoxicity, as revealed by 3-(4,5-dimethylthiazol-2-yl)-2,5-diphenyl tetrazolium bromide (MTT) assays, which showed no significant differences in terms of growth and viability of the cells that were not treated with any oligonucleotides and those cells that were treated with 10–100 nM miR-21 mimics, miR-21 inhibitor, or the control molecules.

### HCV stock preparation

Huh7.5.1 cells were infected with HCV (JFH-1 strain; MOI = 0.1–10) and propagated for 10 days. Stock virus was prepared by collecting and filtering the cell culture supernatant. Aliquots were stored at −80°C until use.

### Isolation and culture of PBMCs

PBMCs were obtained from blood samples diluted with pyrogen-free saline by Histopaque density centrifugation (Haoyang Biotech Co. Ltd.). Cells were washed twice in saline and resuspended in RPMI 1640 with 100 U/ml penicillin and 100 µg/ml streptomycin. PBMCs were then seeded into 6-well plates (1×10^7^/plate), infected with HCV, and incubated at 37°C.

### *In vitro* transcription

RNA was transcribed with a MEGAscriptTM t7 kit (Ambion, Austin, TX, USA) and purified with a MEGS clear kit (Ambion) according to the manufacturer's protocol. FL-J6/JFH-5′C19Rluc2AUbi RNA was extracted by lithium chloride precipitation and quantitated by UV light absorbance. Aliquots of RNA were stored frozen at _80°C for further experiments.

### Luciferase reporter assays

Both the full-length and a series of truncated miPPR-21 fragments were PCR-amplified from 293T genomic DNA using the primers listed in Table S1. The fragments were then digested by KpnI and XhoI and cloned into the KpnI/XhoI site of pGL3-basic (Promega). The mutant miPPR-21 insets were generated by site-directed mutagenesis using specific primers listed in Table S1, and the experiment was performed as previously described [71].

MyD88 and IRAK1 3′ UTR luciferase reporter constructs were generated by cloning PCR-amplified human MyD88 or IRAK1 mRNA 3′ UTR target sites into the XbaI site of pGL3-Basic (Promega). Huh7 cells were cotransfected with the luciferase reporter plasmid (80 ng), pRL-TK Renilla luciferase control plasmid (40 ng), and the indicated RNAs (final concentration, 20 nM). After 24 h, luciferase activities were determined with the Dual-Luciferase Reporter Assay System (Promega) according to the manufacturer's instructions. Data were normalized for transfection efficiency by dividing firefly luciferase activity with that of Renilla luciferase.

### RNA quantification

Total RNA was extracted from cells with TRIzol reagent (Invitrogen) according to the manufacturer's instructions. Quantitative RT-PCR (qPCR) analysis was performed using the Roche LightCycler 480 and SYBR RT-PCR kits (DBI Bioscience); each 20-µl reaction contained 0.5 µM of each PCR primer, 10 µl of SYBR Green PCR master mix, 1 µl of diluted template, and RNase-free water. Primers are shown in Table S2. Target gene expression was normalized by glyceraldehyde 3-phosphate dehydrogenase (GAPDH) expression.

Quantification of miRNAs and Pri-miRNA was performed by qPCR using miRNA analysis kits (Applied Biosystems) according to the manufacturer's instructions. Quantification of pre-miRNA was performed using Qiagen miScripte Precursor Assay (Qiagen, Hilden, Germany) according to the manufacturer's instructions. The relative expression of miRNAs were normalized to that of internal control U6 snRNA within each sample using the 2^−ΔΔCt^ method, and then standardized to the miRNA levels in mock infected or control miRNA treated cells. [72].

### Semiquantitative RT-PCR analysis

Total RNA was isolated from Huh7 cells using TRIzol reagent (Invitrogen), treated with DNase I, and reverse-transcribed with MLV reverse transcriptase (Promega) and random primers (Takara Shuzo, Tokyo, Japan). PCR was performed in 25-µl reactions with the primers shown in Table S3. IFNAR1 and IFNAR2 primers were the same as those used for qPCR (Table S2). Target gene expression was normalized to that of the internal control β-actin. PCR products were analyzed by electrophoresis on 1% agarose gels containing ethidium bromide.

### Enzyme-linked immunosorbent assay (ELISA)

Huh7 cells were seeded into 24-well plates (2×10^5^ cells/well, 0.5 ml volume), incubated overnight, and then transfected as previously described. After 48 h, the cells were infected with HCV for the indicated time periods. IFN-α level in the cell culture medium were measured with an ELISA kit (PBL InterferonSource).

### Chromatin immunoprecipitation (ChIP) and Western blot analysis

ChIP assays were performed using reagents commercially obtained from Upstate, essentially according to the manufacturer's instructions but with the following modifications. Huh7 cells were cross-linked in 1% formaldehyder for 10 min at room temperature. After washing twice with phosphate-buffered saline containing protease inhibitors, the cells were lysed with 200 µL of SDS-lysis buffer and the chromatin/DNA was sonicated to around 500 bp. Immunopreciptatins were performed by incubating sheared chromatin (2×10^6^ cells) overnight at 4°C with 2 µg of antibody followed by binding to 60 µL of salmon sperm DNA/protein G Sepharose beads. The immunopreciptates were washed and eluted in a solution containing of 1% SDS and 0.1 M NaHCO_3_ and incubated overnight at 65°C with 20 µL of 5 M NaCl for reverse cross-linking. DNA was purified with QIAquick columns (Qiagen) and each fragment was PCR-amplified using the primer pairs listed in Table S1.

Whole-cell lysates were prepared by lysing cells with PBS (pH 7.4) containing 0.01% Triton-100, 0.01% EDTA, and 10% cocktail protease inhibitor (Roche). Protein concentration was determined by the Bradford assay (Bio-Rad, Redmond, WA Bio-Rad). Cell lysates (100 µg) were separated by 12% SDS-PAGE and then transferred to a nitrocellulose membrane (Amersham, Bucks, U.K.). The membranes were blocked with 5% nonfat dried milk before incubating with target-specific antibodies. Protein bands were detected with SuperSignal Chemiluminescent reagents (Pierce).

### Flow cytometry

MyD88 and IRAK1 3′ UTR GFP constructs were generated by cloning the PCR-amplified human MyD88 or IRAK1 mRNA 3′ UTR target sites into the XhoI and BamHI sites of pEGFP-C1 (Promega). HEK293 cells were cotransfected with control plasmids (GFP-empty) or GFP-MyD88/IRAK1 3′ UTR plasmids, together with miR-21 mimics or nonspecific RNA oligonucleotides. At 24 h post-transfection, cells were analyzed using a FACSCalibur flow cytometer and CellQuest software (BD Biosciences, San Jose, CA) to determine mean fluorescence intensity and the percentage of cells expressing GFP.

### miRNA mimics and inhibitors

miR-21 mimics (double-stranded RNA oligonucleotides) and miR-21 inhibitors (single-stranded chemically modified oligonucleotides) (final concentration, 50 nM; GuangZhou RiBo Biotech Co. Ltd, GuangZhou, China) were transfected into Huh7 cells for miR-21 overexpression and inhibition, respectively. Nonspecific RNA mimics or inhibitors were transfected as negative controls. They had similar chemical properties as the miR-21 mimics or miR-21 inhibitor, respectively. All miRNAs were purchase from RiboBio (GuangZhou Ribo Biotech Co. Ltd.).

### RNA interference

All siRNAs and the control siRNA (si-Ctrl) were purchased from RiboBio (GuangZhou RiBo Biotech Co. Ltd.).To knock down IRF3, TRIF, HCV, MyD88 and IRAK1, the following siRNAs were used: IRF3, 5′- AGA CAU UCU GGA UGA GUU A-3′ [73]; TRIF, 5′- GAC CAG ACG CCA CTC CAA C -3′ [74]; HCV, 5′-CCT CAA AGA AAA ACC AAA CTT -3′; IRAK1, 5′-AAG UUG CCA UCC UCA GCC UCC-3′ [75]; MyD88, 5′-AAG GCA AUG AAG AAA GAG UUC-3′; nonspecific control sequence, 5′-UUC UCC GAA CGU GUC ACG U-3′ [76]. Transfection was performed with Lipofectamine 2000 (Invitrogen). All siRNAs were tested and verified to reduce expression in Huh7 cells by Western blot analysis (>80% protein reduction) or qPCR (>50% mRNA). Huh7 cells were transfected with 50 nM of each siRNA 48 h before HCV infection.

### HCV yield qualification

Huh7 cells were transfected and infected with HCV as previously described. Viral RNA in the cell culture medium was isolated with the RNApure Virus Kit (CW Biotech), and HCV RNA replicates were quantified by qPCR as previously described.

### Statistical analysis

All experiments were repeated at least three times with similar results. Data were compared by Student's *t-*test. Results are expressed as mean ± SD. *P*<0.05 was considered significant.

### Accession numbers

All the accession numbers/ID numbers for genes and proteins mentioned in the text were listed in Table S4.

## Supporting Information

Figure S1 **The inhibition of HCV reduced miR-21 expression.** (*A and B*) Human Huh7 hepatocytes were infected with or without HCV (MOI = 1) for different times as indicated. The expression of pre-miR21 (*left panel*) and pri-miR21 (*right panel*) (A) and miR-93 (B) was determined by qPCR and normalized to the expression of U6 in each sample. (*C*) Huh7 hepatocytes were incubated with or without UV-irradiated inactive HCV. miR-21 levels were determined by qPCR and normalized to U6 expression. (*D*) Huh7 cells were transfected with FL-J6/JFH5′C19Rluc2AUbi (0.1 µg) and then treated with siRNA-control or siRNA-HCV. Luciferase activities were measured at 48 h posttransfection. (*E*) Huh7 hepatocytes were transfected with or without FL-J6/JFH5′C19Rluc2AUbi (0.1 µg) and then treated with siRNA-control or siRNA-HCV. The miR-21 expression was measured by qPCR. The results are expressed as the mean ± SD (n = 3). The data shown are representative of three independent experiments. **P<0.01.
(TIF)

Figure S2 **AP-1 binding site elements are crucial for miR-21 induction by NS5A and NS3/4A.** Huh7 cells were co-transfected with NS3/4A (*left panel*) or NS5A (*right panel*) and luciferase reporter plasmid specific for the indicated signaling. Luciferase activity was measured. Data are shown as the means SD (n = 3) from one representative experiment. Similar results were obtained in three independent experiments.
(TIF)

Figure S3 **The miR-21 inhibits HCV-triggered production of proinflammatory cytokines and chemokines and activation of the MAPK/ERK pathway.** (A) Huh7 hepatocytes were incubated with or without UV-irradiated inactive HCV. IFN-α mRNA levels (*left*) were determined by qPCR and normalized to GAPDH expression. IFN-α secretion into the cell culture medium (*right*) was measured by ELISA. (*B*) Huh7 hepatocytes were transfected with or without FL-J6/JFH5′C19Rluc2AUbi (0.1 µg). IFN-α mRNA levels (*left*) were determined by qPCR and normalized to the expression of GAPDH in each sample. IFN-α secretion into the cell culture medium (*right*) was measured by ELISA. (*C*) Huh7 cells were transfected with FL-J6/JFH5′C19Rluc2AUbi (0.1 µg). HCV expression was measured by luciferase activity assays at the indicated times. (*D* and *E*) Huh7 hepatocytes (0.5 ml, 2×10^5^ cells) were transfected with miR-21 mimics or control RNA (final concentration, 50 nM). After 48 h, the cells were transfected with FL-J6/JFH5′C19Rluc2AUbi (0.1 µg) for 24 h. The secretion of IL-6 and TNF-α (*D*) and chemokine IL-8 (*E*) into the cell culture medium was determined by ELISA. The results are expressed as the mean ± SD (n = 3). Data are representative of three independent experiments. **P<0.01; *P<0.05. (*F*) Huh7 hepatocytes were transfected as in (*A*) and infected with HCV (MOI = 1) for the indicated time period. NF-κB p65 phosphorylation was detected by immunoblot analysis, using β-actin as a loading control. The blot is a representative of three experiments with similar results.
(TIF)

Figure S4 **The miR-21 attenuates the phosphorylation of STAT1 and STAT2 and expression of PKR, Mx, and OAS.** Huh7 hepatocytes were transfected with control mimics or miR-21 mimics, control inhibitor or miR-21 inhibitor (final concentration, 50 nM), as indicated. After transfection for 30 h, cells were treated with recombinant human IFN-α (100 U/ml) or infected with Sendai virus (SeV). (*A*) After 12 h, p-STAT1, p-STAT2, and total STAT1 and STAT2 were determined by Western blot. (*B*) Huh7 hepatocytes were treated as described above. The PKR, Mx and OAS protein levels were determined by Western blot, using β-actin as a loading control. The results are expressed as the mean ± SD (n = 3). **P<0.01; *P<0.05.
(TIF)

Figure S5
**miR-21 attenuates IRF7 protein expression.** Huh7 hepatocytes were transfected with miR-21 mimics or control RNA, miR-21 inhibitor or control inhibitor (final concentration, 50 nM). After 48 h, IRF7 expression was evaluated by qPCR (*A*), RT-PCR (*B*), and Western blot (*C*). Lamin A was used as a marker for nuclei. The results are expressed as the means
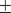
SD (n = 3). **P<0.01; *P<0.05(TIF)

Figure S6
**HCV alone also downregulates components of the Toll-like receptor 7 signaling cascade.** Huh7 hepatocytes were transfected with FL-J6/JFH5′C19Rluc2AUbi (0.1 µg) for 24 h. IRAK1, IRAK4, MyD88, TRAF6 and IRF-7 mRNA levels were determined by qPCR (*A*). The levels of nuclear IRF-7 were determined by Western blot (*B*). MyD88 and IRAK1 protein levels were determined by Western blot and normalized to β-actin (*C*). (*D*)Huh7 cells were transfected with miR-21 inhibitor or control inhibitor followed by HCV infection. The levels of MyD88 and IRAK1 were determined by Western blot. Data are given as the means
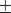
SD (n = 3) from one representative experiment. Similar results were obtained in three independent experiments.*, p<0.05.(TIF)

Figure S7
**miR-21 targets human MyD88 and IRAK1.** (*A*) Mutated sequence of the miR-21 binding site with the IRAK1 and MyD88 3′UTR. (*B*) The effect of miR-21 (*upper panel*) or miR-21 inhibitor (*lowe*r panel) on the luciferase activity of reporter vectors with mutant IRAK1 and MyD88 3′UTR. (*C*) The effect of miR-21 on the GFP activity of reporter vectors with mutant IRAK1 and MyD88 3′UTR. The results are expressed as the means
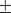
SD (n = 3). black triangle, p>0.05.(TIF)

Figure S8
**The miR-21-mediated regulation of the IFN-α pathway is independent of the TRIF pathway.** Huh7 cells were transfected with FL-J6/JFH5′C19Rluc2AUbi (0.1 µg) and treated with siRNA, as indicated, for 12 h. The secretion of IFN-α into the cell culture medium was measured by ELISA. Data are given as the means
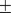
SD (n = 3) from one representative experiment. Similar results were obtained in three independent experiments. *, p<0.05; black triangle, p>0.05.(TIF)

Figure S9 **The expression level of NS5A protein during pCMV-NS5A transfection or HCV infection.** Huh7 cells were transfected or infected with pCMV-NS5A (*left panel*) or HCV (MOI = 1) (*right panel*) for different times as indicated, respectively. The levels of NS5A were determined by Western blot and normalized to β-actin. Similar results were obtained in three independent experiments.
(TIF)

Figure S10 **The localization of NS5A and NS3/4A protein in various cell types.** Huh7 cells (*upper panel*) were transfected with pCMV-NS5A, pCMV-NS3/4A, or control vector as indicated, respectively, for 48 h. After fixation, the cells were immunostained with antibody for Flag. The nuclei were stained by DAPI. The L02 (*middle panel*) and 293 (*lower panel*) cells were transfected and treated as Huh7 cells. Similar results were obtained in three independent experiments.
(TIF)

Table S1 **Primers and oligonucleotide sequences in the study**
(DOC)

Table S2 **Primers used for qPCR.**
(DOC)

Table S3 **Primers used for semiquantitative RT-PCR.**
(DOC)

Table S4 **Accession numbers.**
(DOC)

## References

[1] Houghton M, Weiner A, Han J, Kuo G, Choo QL (1991) Molecular biology of the hepatitis C viruses: implications for diagnosis, development and control of viral disease. Hepatology 14: 381–388. 1650328

[2] Robertson B, Myers G, Howard C, Brettin T, Bukh J, et al (1998) Classification, nomenclature, and database development for hepatitis C virus (HCV) and related viruses: proposals for standardization. International Committee on Virus Taxonomy. Arch Virol 143: 2493–2503. 9930205 10.1007/s007050050479

[3] Choo QL, Kuo G, Weiner AJ, Overby LR, Bradley DW, et al (1989) Isolation of a cDNA clone derived from a blood-borne non-A, non-B viral hepatitis genome. Science 244: 359–362. 2523562 10.1126/science.2523562

[4] Lindenbach BD, Evans MJ, Syder AJ, Wolk B, Tellinghuisen TL, et al (2005) Complete replication of hepatitis C virus in cell culture. Science 309: 623–626. 15947137 10.1126/science.1114016

[5] Lauer GM, Walker BD (2001) Hepatitis C virus infection. N Engl J Med 345: 41–52. 11439948 10.1056/NEJM200107053450107

[6] Akira S, Uematsu S, Takeuchi O (2006) Pathogen recognition and innate immunity. Cell 124: 783–801. 16497588 10.1016/j.cell.2006.02.015

[7] Beutler B, Eidenschenk C, Crozat K, Imler JL, Takeuchi O, et al (2007) Genetic analysis of resistance to viral infection. Nat Rev Immunol 7: 753–766. 17893693 10.1038/nri2174

[8] Liew FY, Xu D, Brint EK, O'Neill LA (2005) Negative regulation of toll-like receptor-mediated immune responses. Nat Rev Immunol 5: 446–458. 15928677 10.1038/nri1630

[9] Jackson RJ, Standart N (2007) How do microRNAs regulate gene expression? Sci STKE 2007: re1. 17200520 10.1126/stke.3672007re1

[10] Wienholds E, Plasterk RH (2005) MicroRNA function in animal development. FEBS Lett 579: 5911–5922. 16111679 10.1016/j.febslet.2005.07.070

[11] Chen JF, Mandel EM, Thomson JM, Wu Q, Callis TE, et al (2006) The role of microRNA-1 and microRNA-133 in skeletal muscle proliferation and differentiation. Nat Genet 38: 228–233. 16380711 10.1038/ng1725 PMC2538576

[12] Cimmino A, Calin GA, Fabbri M, Iorio MV, Ferracin M, et al (2005) miR-15 and miR-16 induce apoptosis by targeting BCL2. Proc Natl Acad Sci U S A 102: 13944–13949. 16166262 10.1073/pnas.0506654102 PMC1236577

[13] Brennecke J, Hipfner DR, Stark A, Russell RB, Cohen SM (2003) bantam encodes a developmentally regulated microRNA that controls cell proliferation and regulates the proapoptotic gene hid in Drosophila. Cell 113: 25–36. 12679032 10.1016/s0092-8674(03)00231-9

[14] Hatfield SD, Shcherbata HR, Fischer KA, Nakahara K, Carthew RW, et al (2005) Stem cell division is regulated by the microRNA pathway. Nature 435: 974–978. 15944714 10.1038/nature03816

[15] Hou J, Wang P, Lin L, Liu X, Ma F, et al (2009) MicroRNA-146a feedback inhibits RIG-I-dependent Type I IFN production in macrophages by targeting TRAF6, IRAK1, and IRAK2. J Immunol 183: 2150–2158. 19596990 10.4049/jimmunol.0900707

[16] O'Connell RM, Taganov KD, Boldin MP, Cheng G, Baltimore D (2007) MicroRNA-155 is induced during the macrophage inflammatory response. Proc Natl Acad Sci U S A 104: 1604–1609. 17242365 10.1073/pnas.0610731104 PMC1780072

[17] Vigorito E, Perks KL, Abreu-Goodger C, Bunting S, Xiang Z, et al (2007) microRNA-155 regulates the generation of immunoglobulin class-switched plasma cells. Immunity 27: 847–859. 18055230 10.1016/j.immuni.2007.10.009 PMC4135426

[18] Thai TH, Calado DP, Casola S, Ansel KM, Xiao C, et al (2007) Regulation of the germinal center response by microRNA-155. Science 316: 604–608. 17463289 10.1126/science.1141229

[19] Hu G, Zhou R, Liu J, Gong AY, Chen XM MicroRNA-98 and let-7 regulate expression of suppressor of cytokine signaling 4 in biliary epithelial cells in response to Cryptosporidium parvum infection. J Infect Dis 202: 125–135. 20486857 10.1086/653212 PMC2880649

[20] Taganov KD, Boldin MP, Baltimore D (2007) MicroRNAs and immunity: tiny players in a big field. Immunity 26: 133–137. 17307699 10.1016/j.immuni.2007.02.005

[21] Baltimore D, Boldin MP, O'Connell RM, Rao DS, Taganov KD (2008) MicroRNAs: new regulators of immune cell development and function. Nat Immunol 9: 839–845. 18645592 10.1038/ni.f.209

[22] Lodish HF, Zhou B, Liu G, Chen CZ (2008) Micromanagement of the immune system by microRNAs. Nat Rev Immunol 8: 120–130. 18204468 10.1038/nri2252

[23] Jiang J, Gusev Y, Aderca I, Mettler TA, Nagorney DM, et al (2008) Association of MicroRNA expression in hepatocellular carcinomas with hepatitis infection, cirrhosis, and patient survival. Clin Cancer Res 14: 419–427. 18223217 10.1158/1078-0432.CCR-07-0523 PMC2755230

[24] Meng F, Henson R, Wehbe-Janek H, Ghoshal K, Jacob ST, et al (2007) MicroRNA-21 regulates expression of the PTEN tumor suppressor gene in human hepatocellular cancer. Gastroenterology 133: 647–658. 17681183 10.1053/j.gastro.2007.05.022 PMC4285346

[25] Peng X, Li Y, Walters KA, Rosenzweig ER, Lederer SL, et al (2009) Computational identification of hepatitis C virus associated microRNA-mRNA regulatory modules in human livers. BMC Genomics 10: 373. 19671175 10.1186/1471-2164-10-373 PMC2907698

[26] Fujita S, Ito T, Mizutani T, Minoguchi S, Yamamichi N, et al (2008) miR-21 Gene expression triggered by AP-1 is sustained through a double-negative feedback mechanism. J Mol Biol 378: 492–504. 18384814 10.1016/j.jmb.2008.03.015

[27] Toker A (1998) Signaling through protein kinase C. Front Biosci 3: D1134–1147. 9792904 10.2741/a350

[28] Black JD (2000) Protein kinase C-mediated regulation of the cell cycle. Front Biosci 5: D406–423. 10762593 10.2741/black

[29] Storz P, Doppler H, Toker A (2004) Protein kinase Cdelta selectively regulates protein kinase D-dependent activation of NF-kappaB in oxidative stress signaling. Mol Cell Biol 24: 2614–2626. 15024053 10.1128/MCB.24.7.2614-2626.2004 PMC371115

[30] Liu M, Yang Y, Gu C, Yue Y, Wu KK, et al (2007) Spike protein of SARS-CoV stimulates cyclooxygenase-2 expression via both calcium-dependent and calcium-independent protein kinase C pathways. FASEB J 21: 1586–1596. 17267381 10.1096/fj.06-6589com

[31] Lu L, Wei L, Peng G, Mu Y, Wu K, et al (2008) NS3 protein of hepatitis C virus regulates cyclooxygenase-2 expression through multiple signaling pathways. Virology 371: 61–70. 17964630 10.1016/j.virol.2007.09.025 PMC7103338

[32] Krutzfeldt J, Rajewsky N, Braich R, Rajeev KG, Tuschl T, et al (2005) Silencing of microRNAs in vivo with ‘antagomirs’. Nature 438: 685–689. 16258535 10.1038/nature04303

[33] Samuel CE (2001) Antiviral actions of interferons. Clin Microbiol Rev 14: table of contents, 778–809. 10.1128/CMR.14.4.778-809.2001 PMC89003 11585785

[34] Jouanguy E, Zhang SY, Chapgier A, Sancho-Shimizu V, Puel A, et al (2007) Human primary immunodeficiencies of type I interferons. Biochimie 89: 878–883. 17561326 10.1016/j.biochi.2007.04.016

[35] Dupuis S, Jouanguy E, Al-Hajjar S, Fieschi C, Al-Mohsen IZ, et al (2003) Impaired response to interferon-alpha/beta and lethal viral disease in human STAT1 deficiency. Nat Genet 33: 388–391. 12590259 10.1038/ng1097

[36] de Veer MJ, Holko M, Frevel M, Walker E, Der S, et al (2001) Functional classification of interferon-stimulated genes identified using microarrays. J Leukoc Biol 69: 912–920. 11404376

[37] Samuel CE (2002) Host genetic variability and West Nile virus susceptibility. Proc Natl Acad Sci U S A 99: 11555–11557. 12192094 10.1073/pnas.202448899 PMC129304

[38] Castelli J, Wood KA, Youle RJ (1998) The 2–5A system in viral infection and apoptosis. Biomed Pharmacother 52: 386–390. 9856285 10.1016/s0753-3322(99)80006-7

[39] Huang JT, Schneider RJ (1991) Adenovirus inhibition of cellular protein synthesis involves inactivation of cap-binding protein. Cell 65: 271–280. 1849798 10.1016/0092-8674(91)90161-q

[40] Weber F (2007) Interaction of hepatitis C virus with the type I interferon system. World J Gastroenterol 13: 4818–4823. 17828812 10.3748/wjg.v13.i36.4818 PMC4611759

[41] Lee J, Wu CC, Lee KJ, Chuang TH, Katakura K, et al (2006) Activation of anti-hepatitis C virus responses via Toll-like receptor 7. Proc Natl Acad Sci U S A 103: 1828–1833. 16446426 10.1073/pnas.0510801103 PMC1413670

[42] Judge AD, Sood V, Shaw JR, Fang D, McClintock K, et al (2005) Sequence-dependent stimulation of the mammalian innate immune response by synthetic siRNA. Nat Biotechnol 23: 457–462. 15778705 10.1038/nbt1081

[43] Smith MW, Yue ZN, Korth MJ, Do HA, Boix L, et al (2003) Hepatitis C virus and liver disease: global transcriptional profiling and identification of potential markers. Hepatology 38: 1458–1467. 14647057 10.1016/j.hep.2003.09.024

[44] Honda K, Yanai H, Negishi H, Asagiri M, Sato M, et al (2005) IRF-7 is the master regulator of type-I interferon-dependent immune responses. Nature 434: 772–777. 15800576 10.1038/nature03464

[45] Foy E, Li K, Sumpter R Jr, Loo YM, Johnson CL, et al (2005) Control of antiviral defenses through hepatitis C virus disruption of retinoic acid-inducible gene-I signaling. Proc Natl Acad Sci U S A 102: 2986–2991. 15710892 10.1073/pnas.0408707102 PMC549461

[46] Katze MG, He Y, Gale M Jr (2002) Viruses and interferon: a fight for supremacy. Nat Rev Immunol 2: 675–687. 12209136 10.1038/nri888

[47] Gale M Jr (2003) Effector genes of interferon action against hepatitis C virus. Hepatology 37: 975–978. 12717377 10.1053/jhep.2003.50201

[48] Abe T, Kaname Y, Hamamoto I, Tsuda Y, Wen X, et al (2007) Hepatitis C virus nonstructural protein 5A modulates the toll-like receptor-MyD88-dependent signaling pathway in macrophage cell lines. J Virol 81: 8953–8966. 17567694 10.1128/JVI.00649-07 PMC1951400

[49] Cheng AM, Byrom MW, Shelton J, Ford LP (2005) Antisense inhibition of human miRNAs and indications for an involvement of miRNA in cell growth and apoptosis. Nucleic Acids Res 33: 1290–1297. 15741182 10.1093/nar/gki200 PMC552951

[50] Meng F, Henson R, Lang M, Wehbe H, Maheshwari S, et al (2006) Involvement of human micro-RNA in growth and response to chemotherapy in human cholangiocarcinoma cell lines. Gastroenterology 130: 2113–2129. 16762633 10.1053/j.gastro.2006.02.057

[51] Chan JA, Krichevsky AM, Kosik KS (2005) MicroRNA-21 is an antiapoptotic factor in human glioblastoma cells. Cancer Res 65: 6029–6033. 16024602 10.1158/0008-5472.CAN-05-0137

[52] Frelin L, Brenndorfer ED, Ahlen G, Weiland M, Hultgren C, et al (2006) The hepatitis C virus and immune evasion: non-structural 3/4A transgenic mice are resistant to lethal tumour necrosis factor alpha mediated liver disease. Gut 55: 1475–1483. 16527836 10.1136/gut.2005.085050 PMC1856439

[53] Gosert R, Egger D, Lohmann V, Bartenschlager R, Blum HE, et al (2003) Identification of the hepatitis C virus RNA replication complex in Huh-7 cells harboring subgenomic replicons. J Virol 77: 5487–5492. 12692249 10.1128/JVI.77.9.5487-5492.2003 PMC153965

[54] Ide Y, Zhang L, Chen M, Inchauspe G, Bahl C, et al (1996) Characterization of the nuclear localization signal and subcellular distribution of hepatitis C virus nonstructural protein NS5A. Gene 182: 203–211. 8982089 10.1016/s0378-1119(96)00555-0

[55] Pan W, Zhu S, Yuan M, Cui H, Wang L, et al MicroRNA-21 and microRNA-148a contribute to DNA hypomethylation in lupus CD4+ T cells by directly and indirectly targeting DNA methyltransferase 1. J Immunol 184: 6773–6781. 20483747 10.4049/jimmunol.0904060

[56] Sheedy FJ, Palsson-McDermott E, Hennessy EJ, Martin C, O'Leary JJ, et al Negative regulation of TLR4 via targeting of the proinflammatory tumor suppressor PDCD4 by the microRNA miR-21. Nat Immunol 11: 141–147. 19946272 10.1038/ni.1828

[57] Sayed D, He M, Hong C, Gao S, Rane S, et al MicroRNA-21 is a downstream effector of AKT that mediates its antiapoptotic effects via suppression of Fas ligand. J Biol Chem 285: 20281–20290. 10.1074/jbc.M110.109207 PMC2888441 20404348

[58] Randall G, Panis M, Cooper JD, Tellinghuisen TL, Sukhodolets KE, et al (2007) Cellular cofactors affecting hepatitis C virus infection and replication. Proc Natl Acad Sci U S A 104: 12884–12889. 17616579 10.1073/pnas.0704894104 PMC1937561

[59] Liu X, Wang T, Wakita T, Yang W (2010) Systematic identification of microRNA and messenger RNA profiles in hepatitis C virus-infected human hepatoma cells. Virology 398: 57–67. 20006370 10.1016/j.virol.2009.11.036

[60] Wang C, Pflugheber J, Sumpter R Jr, Sodora DL, Hui D, et al (2003) Alpha interferon induces distinct translational control programs to suppress hepatitis C virus RNA replication. J Virol 77: 3898–3912. 12634350 10.1128/JVI.77.7.3898-3912.2003 PMC150642

[61] Shimazaki T, Honda M, Kaneko S, Kobayashi K (2002) Inhibition of internal ribosomal entry site-directed translation of HCV by recombinant IFN-alpha correlates with a reduced La protein. Hepatology 35: 199–208. 11786977 10.1053/jhep.2002.30202

[62] Guo JT, Bichko VV, Seeger C (2001) Effect of alpha interferon on the hepatitis C virus replicon. J Virol 75: 8516–8523. 11507197 10.1128/JVI.75.18.8516-8523.2001 PMC115097

[63] Dobrovolskaia MA, Medvedev AE, Thomas KE, Cuesta N, Toshchakov V, et al (2003) Induction of in vitro reprogramming by Toll-like receptor (TLR)2 and TLR4 agonists in murine macrophages: effects of TLR “homotolerance” versus “heterotolerance” on NF-kappa B signaling pathway components. J Immunol 170: 508–519. 12496438 10.4049/jimmunol.170.1.508

[64] Takeda K, Kaisho T, Akira S (2003) Toll-like receptors. Annu Rev Immunol 21: 335–376. 12524386 10.1146/annurev.immunol.21.120601.141126

[65] Schlender J, Hornung V, Finke S, Gunthner-Biller M, Marozin S, et al (2005) Inhibition of toll-like receptor 7- and 9-mediated alpha/beta interferon production in human plasmacytoid dendritic cells by respiratory syncytial virus and measles virus. J Virol 79: 5507–5515. 15827165 10.1128/JVI.79.9.5507-5515.2005 PMC1082779

[66] Lei X, Liu X, Ma Y, Sun Z, Yang Y, et al (2010) The 3C protein of enterovirus 71 inhibits retinoid acid-inducible gene I-mediated interferon regulatory factor 3 activation and type I interferon responses. J Virol 84: 8051–8061. 20519382 10.1128/JVI.02491-09 PMC2916543

[67] Selbach M, Schwanhausser B, Thierfelder N, Fang Z, Khanin R, et al (2008) Widespread changes in protein synthesis induced by microRNAs. Nature 455: 58–63. 18668040 10.1038/nature07228

[68] Li K, Foy E, Ferreon JC, Nakamura M, Ferreon AC, et al (2005) Immune evasion by hepatitis C virus NS3/4A protease-mediated cleavage of the Toll-like receptor 3 adaptor protein TRIF. Proc Natl Acad Sci U S A 102: 2992–2997. 15710891 10.1073/pnas.0408824102 PMC548795

[69] Tscherne DM, Jones CT, Evans MJ, Lindenbach BD, McKeating JA, et al (2006) Time- and temperature-dependent activation of hepatitis C virus for low-pH-triggered entry. J Virol 80: 1734–1741. 16439530 10.1128/JVI.80.4.1734-1741.2006 PMC1367161

[70] Liu S, Hao Q, Peng N, Yue X, Wang Y, et al (2012) Major vault protein: a virus-induced host factor against viral replication through the induction of type-I interferon. Hepatology 56: 57–66. 22318991 10.1002/hep.25642

[71] Chen Y, Shen A, Rider PJ, Yu Y, Wu K, et al (2011) A liver-specific microRNA binds to a highly conserved RNA sequence of hepatitis B virus and negatively regulates viral gene expression and replication. FASEB J 25: 4511–4521. 21903935 10.1096/fj.11-187781 PMC3236624

[72] Livak KJ, Schmittgen TD (2001) Analysis of relative gene expression data using real-time quantitative PCR and the 2(−Delta Delta C(T)) Method. Methods 25: 402–408. 11846609 10.1006/meth.2001.1262

[73] Myskiw C, Arsenio J, van Bruggen R, Deschambault Y, Cao J (2009) Vaccinia virus E3 suppresses expression of diverse cytokines through inhibition of the PKR, NF-kappaB, and IRF3 pathways. J Virol 83: 6757–6768. 19369349 10.1128/JVI.02570-08 PMC2698532

[74] Oshiumi H, Matsumoto M, Funami K, Akazawa T, Seya T (2003) TICAM-1, an adaptor molecule that participates in Toll-like receptor 3-mediated interferon-beta induction. Nat Immunol 4: 161–167. 12539043 10.1038/ni886

[75] Wan J, Sun L, Mendoza JW, Chui YL, Huang DP, et al (2004) Elucidation of the c-Jun N-terminal kinase pathway mediated by Estein-Barr virus-encoded latent membrane protein 1. Mol Cell Biol 24: 192–199. 14673155 10.1128/MCB.24.1.192-199.2004 PMC303354

[76] Hirata Y, Ohmae T, Shibata W, Maeda S, Ogura K, et al (2006) MyD88 and TNF receptor-associated factor 6 are critical signal transducers in Helicobacter pylori-infected human epithelial cells. J Immunol 176: 3796–3803. 16517750 10.4049/jimmunol.176.6.3796

