# Supplementary figures and images for: HCV-Induced miR-21 Contributes to Evasion of Host Immune System by Targeting MyD88 and IRAK1
Source: PLoS Pathog. 2013 Apr 25;9(4):e1003248. doi: 10.1371/journal.ppat.1003248 (PMC3635988; doi:10.1371/journal.ppat.1003248)

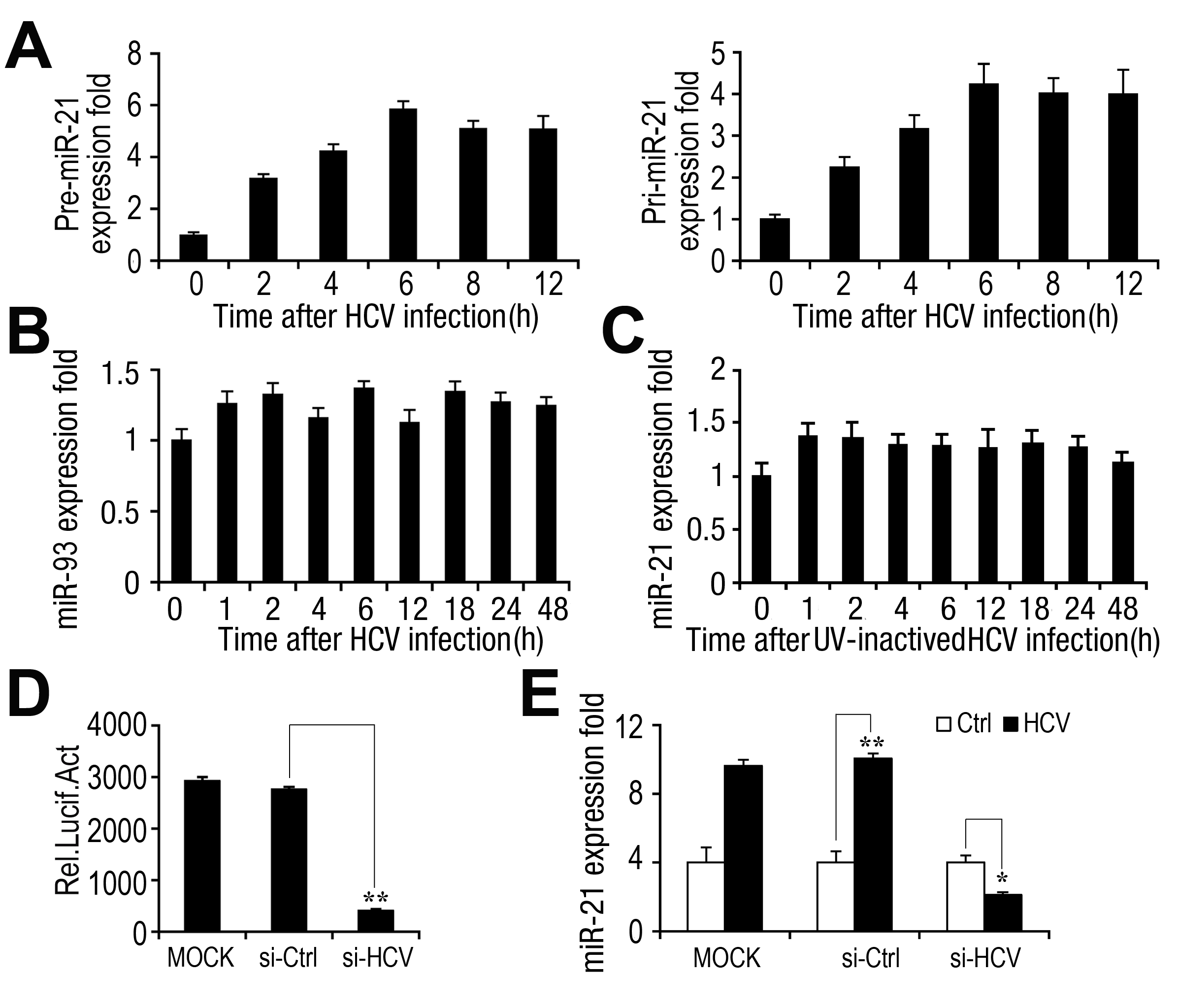

Supplement: Figure S1 — The inhibition of HCV reduced miR-21 expression. (A and B) Human Huh7 hepatocytes were infected with or without HCV (MOI = 1) for different times as indicated. The expression of pre-miR21 (left panel) and pri-miR21 (right panel) (A) and miR-93 (B) was determined by qPCR and normalized to the expression of U6 in each sample. (C) Huh7 hepatocytes were incubated with or without UV-irradiated inactive HCV. miR-21 levels were determined by qPCR and normalized to U6 expression. (D) Huh7 cells were transfected with FL-J6/JFH5′C19Rluc2AUbi (0.1 µg) and then treated with siRNA-control or siRNA-HCV. Luciferase activities were measured at 48 h posttransfection. (E) Huh7 hepatocytes were transfected with or without FL-J6/JFH5′C19Rluc2AUbi (0.1 µg) and then treated with siRNA-control or siRNA-HCV. The miR-21 expression was measured by qPCR. The results are expressed as the mean ± SD (n = 3). The data shown are representative of three independent experiments. **P<0.01. (TIF) [file ppat.1003248.s001.tif]

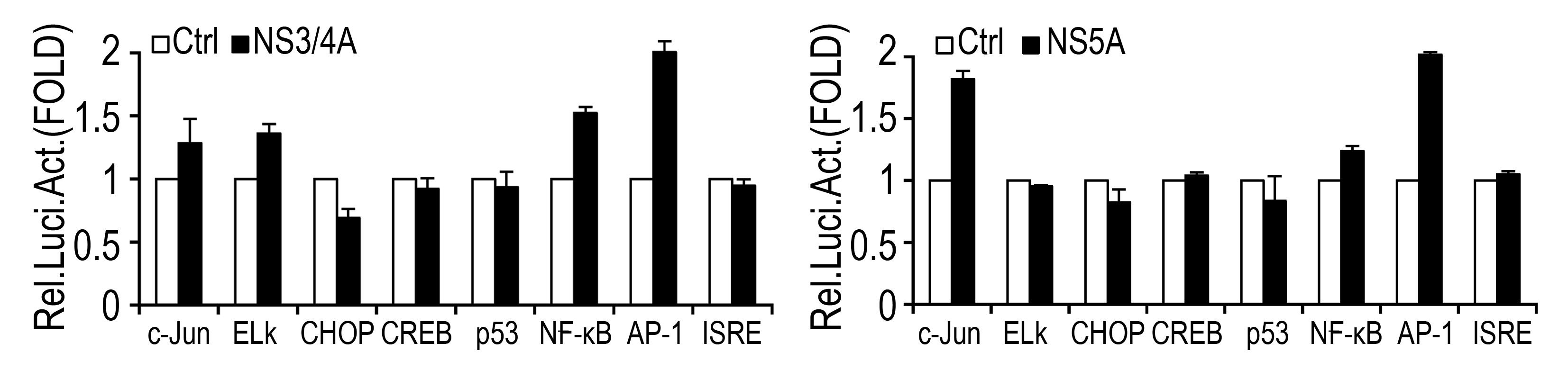

Supplement: Figure S2 — AP-1 binding site elements are crucial for miR-21 induction by NS5A and NS3/4A. Huh7 cells were co-transfected with NS3/4A (left panel) or NS5A (right panel) and luciferase reporter plasmid specific for the indicated signaling. Luciferase activity was measured. Data are shown as the means SD (n = 3) from one representative experiment. Similar results were obtained in three independent experiments. (TIF) [file ppat.1003248.s002.tif]

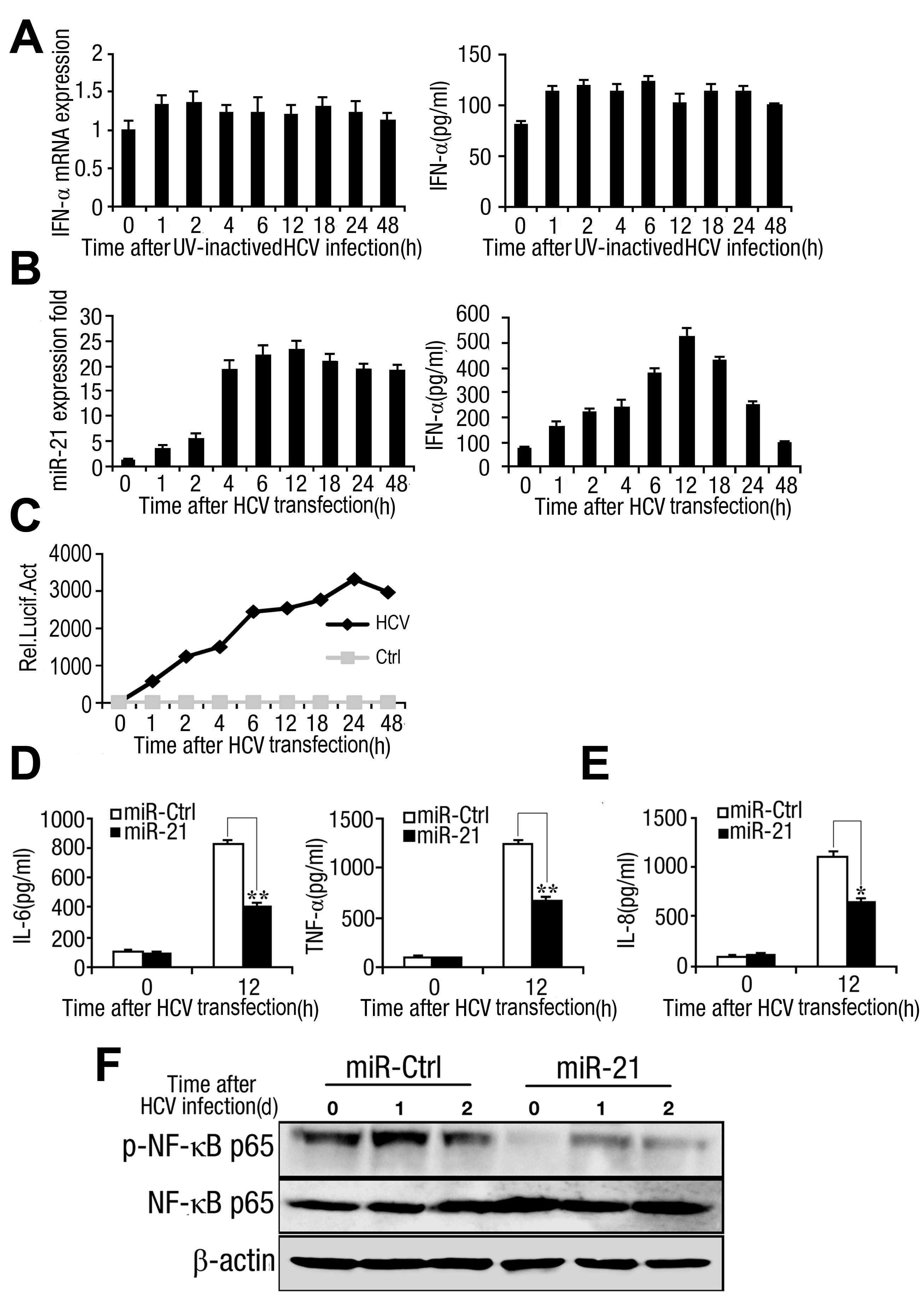

Supplement: Figure S3 — The miR-21 inhibits HCV-triggered production of proinflammatory cytokines and chemokines and activation of the MAPK/ERK pathway. (A) Huh7 hepatocytes were incubated with or without UV-irradiated inactive HCV. IFN-α mRNA levels (left) were determined by qPCR and normalized to GAPDH expression. IFN-α secretion into the cell culture medium (right) was measured by ELISA. (B) Huh7 hepatocytes were transfected with or without FL-J6/JFH5′C19Rluc2AUbi (0.1 µg). IFN-α mRNA levels (left) were determined by qPCR and normalized to the expression of GAPDH in each sample. IFN-α secretion into the cell culture medium (right) was measured by ELISA. (C) Huh7 cells were transfected with FL-J6/JFH5′C19Rluc2AUbi (0.1 µg). HCV expression was measured by luciferase activity assays at the indicated times. (D and E) Huh7 hepatocytes (0.5 ml, 2×10^5^ cells) were transfected with miR-21 mimics or control RNA (final concentration, 50 nM). After 48 h, the cells were transfected with FL-J6/JFH5′C19Rluc2AUbi (0.1 µg) for 24 h. The secretion of IL-6 and TNF-α (D) and chemokine IL-8 (E) into the cell culture medium was determined by ELISA. The results are expressed as the mean ± SD (n = 3). Data are representative of three independent experiments. **P<0.01; *P<0.05. (F) Huh7 hepatocytes were transfected as in (A) and infected with HCV (MOI = 1) for the indicated time period. NF-κB p65 phosphorylation was detected by immunoblot analysis, using β-actin as a loading control. The blot is a representative of three experiments with similar results. (TIF) [file ppat.1003248.s003.tif]

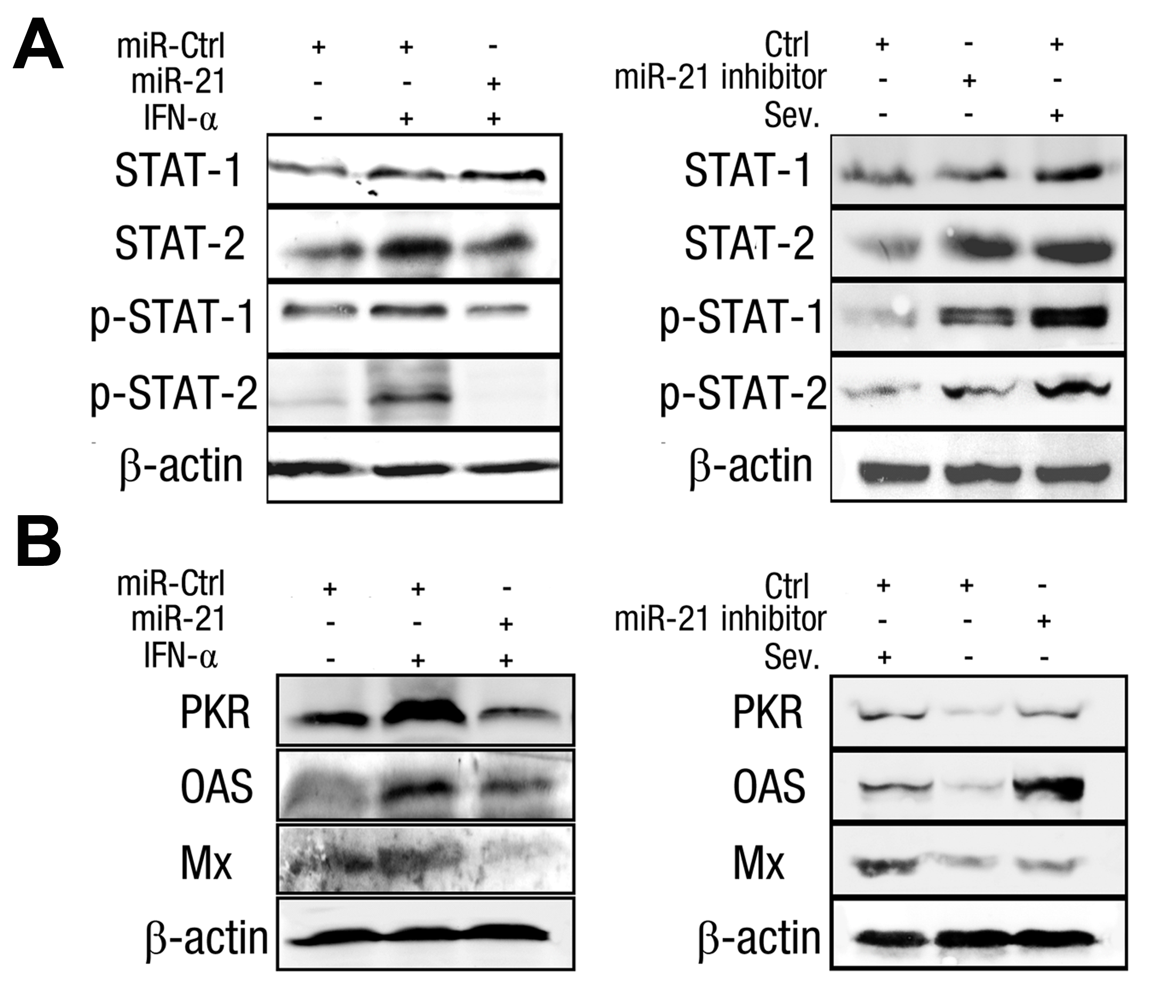

Supplement: Figure S4 — The miR-21 attenuates the phosphorylation of STAT1 and STAT2 and expression of PKR, Mx, and OAS. Huh7 hepatocytes were transfected with control mimics or miR-21 mimics, control inhibitor or miR-21 inhibitor (final concentration, 50 nM), as indicated. After transfection for 30 h, cells were treated with recombinant human IFN-α (100 U/ml) or infected with Sendai virus (SeV). (A) After 12 h, p-STAT1, p-STAT2, and total STAT1 and STAT2 were determined by Western blot. (B) Huh7 hepatocytes were treated as described above. The PKR, Mx and OAS protein levels were determined by Western blot, using β-actin as a loading control. The results are expressed as the mean ± SD (n = 3). **P<0.01; *P<0.05. (TIF) [file ppat.1003248.s004.tif]

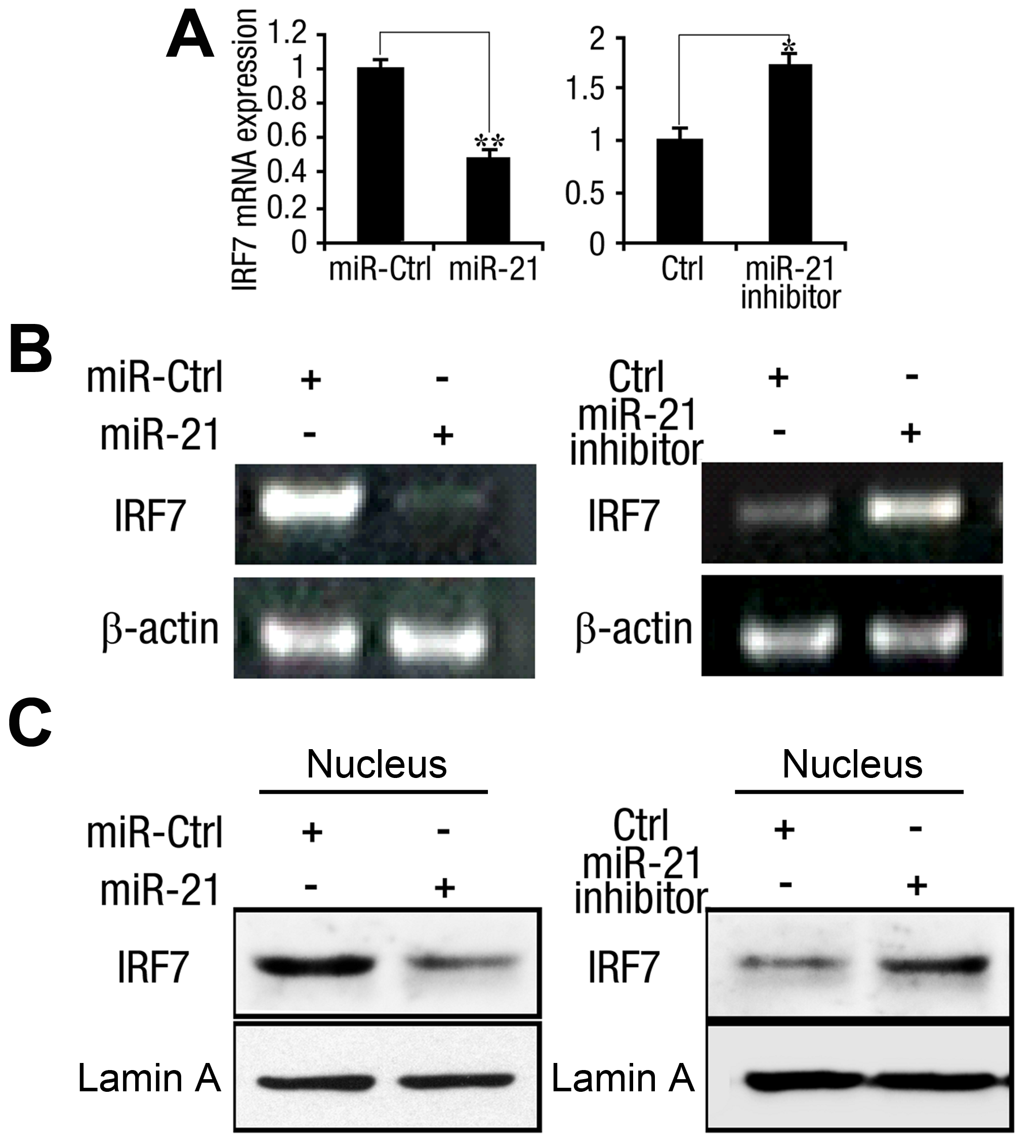

Supplement: Figure S5 — miR-21 attenuates IRF7 protein expression. Huh7 hepatocytes were transfected with miR-21 mimics or control RNA, miR-21 inhibitor or control inhibitor (final concentration, 50 nM). After 48 h, IRF7 expression was evaluated by qPCR (A), RT-PCR (B), and Western blot (C). Lamin A was used as a marker for nuclei. The results are expressed as the meansSD (n = 3). **P<0.01; *P<0.05 (TIF) [file ppat.1003248.s005.tif]

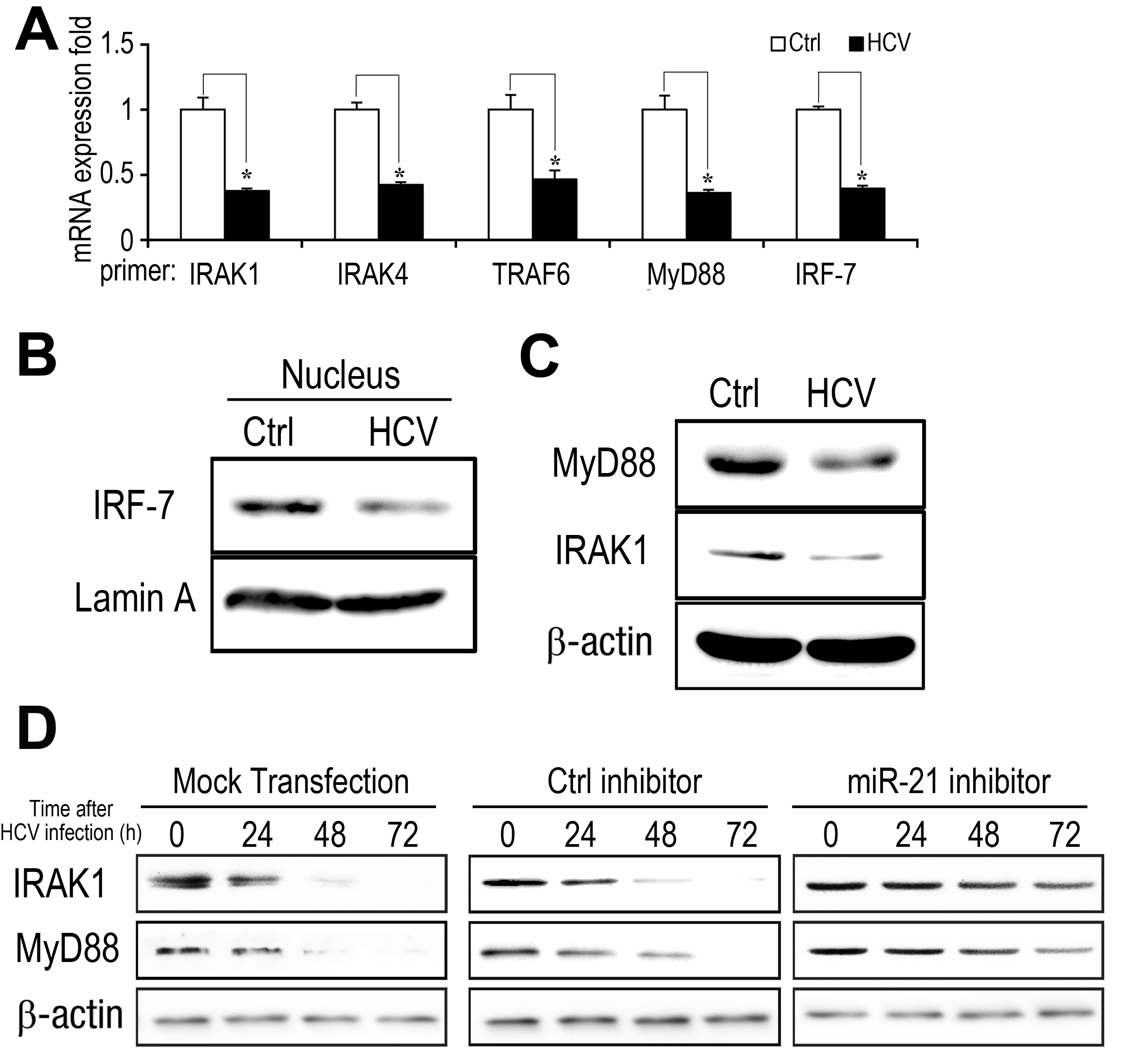

Supplement: Figure S6 — HCV alone also downregulates components of the Toll-like receptor 7 signaling cascade. Huh7 hepatocytes were transfected with FL-J6/JFH5′C19Rluc2AUbi (0.1 µg) for 24 h. IRAK1, IRAK4, MyD88, TRAF6 and IRF-7 mRNA levels were determined by qPCR (A). The levels of nuclear IRF-7 were determined by Western blot (B). MyD88 and IRAK1 protein levels were determined by Western blot and normalized to β-actin (C). (D)Huh7 cells were transfected with miR-21 inhibitor or control inhibitor followed by HCV infection. The levels of MyD88 and IRAK1 were determined by Western blot. Data are given as the meansSD (n = 3) from one representative experiment. Similar results were obtained in three independent experiments.*, p<0.05. (TIF) [file ppat.1003248.s006.tif]

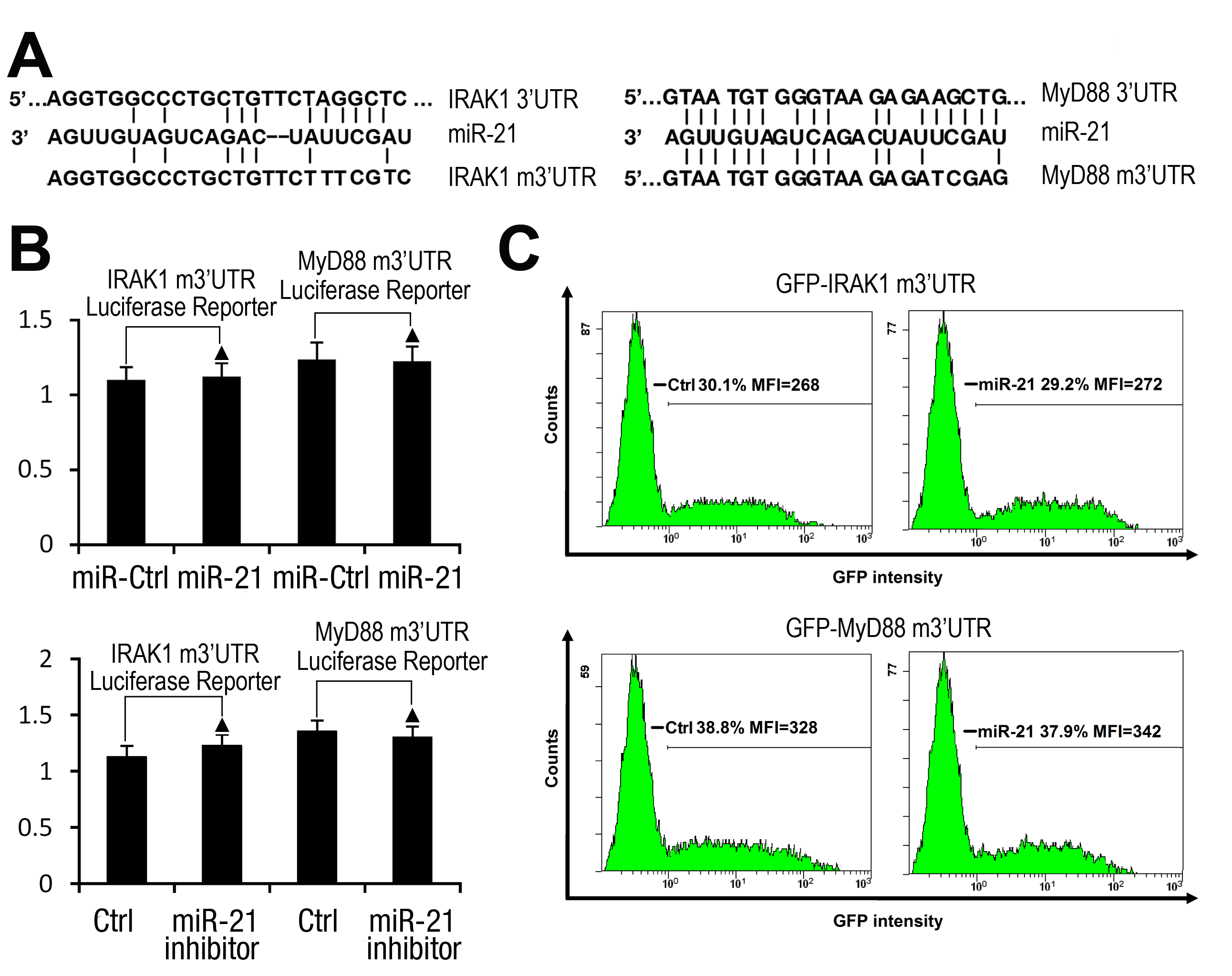

Supplement: Figure S7 — miR-21 targets human MyD88 and IRAK1. (A) Mutated sequence of the miR-21 binding site with the IRAK1 and MyD88 3′UTR. (B) The effect of miR-21 (upper panel) or miR-21 inhibitor (lower panel) on the luciferase activity of reporter vectors with mutant IRAK1 and MyD88 3′UTR. (C) The effect of miR-21 on the GFP activity of reporter vectors with mutant IRAK1 and MyD88 3′UTR. The results are expressed as the meansSD (n = 3). black triangle, p>0.05. (TIF) [file ppat.1003248.s007.tif]

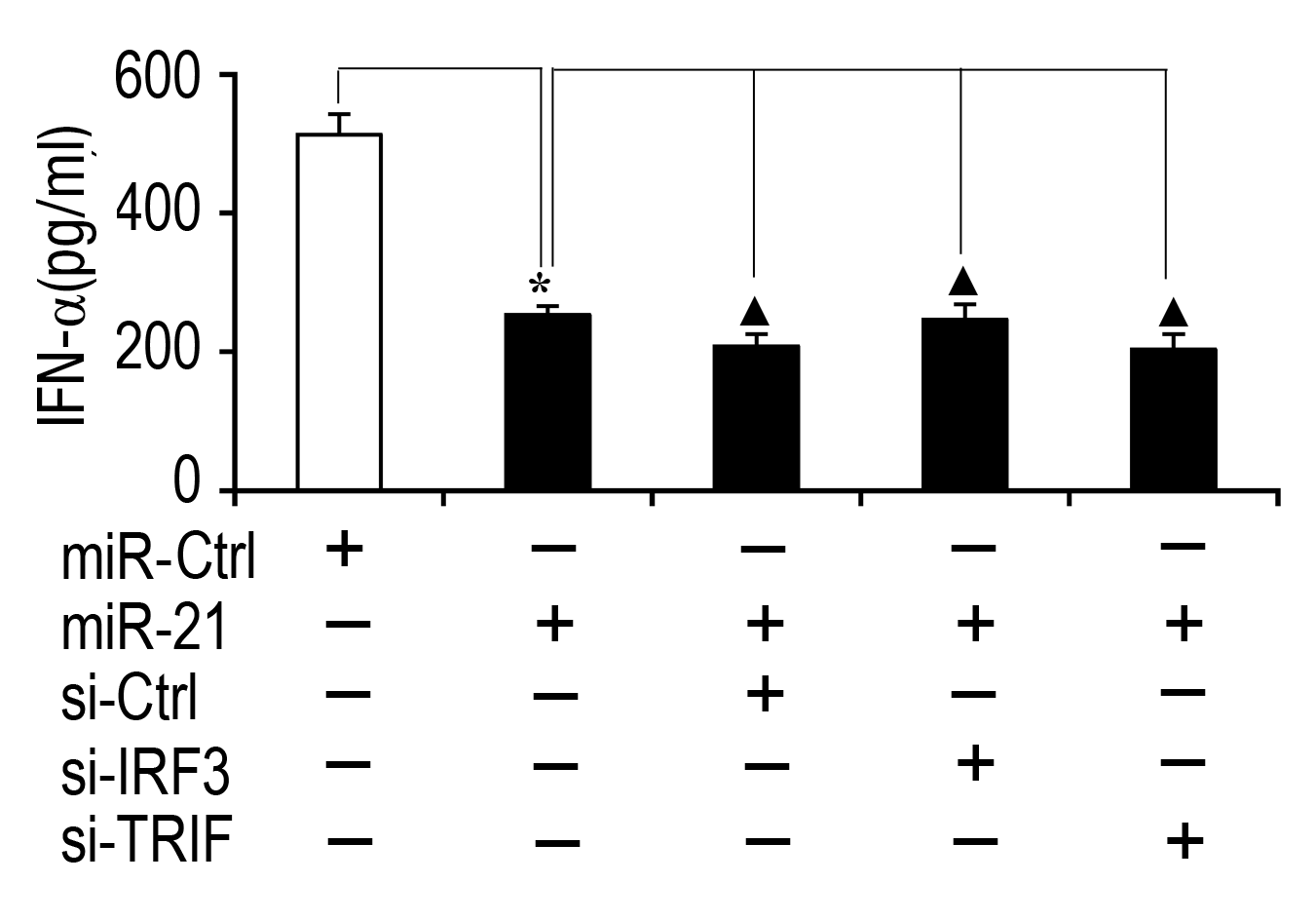

Supplement: Figure S8 — The miR-21-mediated regulation of the IFN-α pathway is independent of the TRIF pathway. Huh7 cells were transfected with FL-J6/JFH5′C19Rluc2AUbi (0.1 µg) and treated with siRNA, as indicated, for 12 h. The secretion of IFN-α into the cell culture medium was measured by ELISA. Data are given as the meansSD (n = 3) from one representative experiment. Similar results were obtained in three independent experiments. *, p<0.05; black triangle, p>0.05. (TIF) [file ppat.1003248.s008.tif]

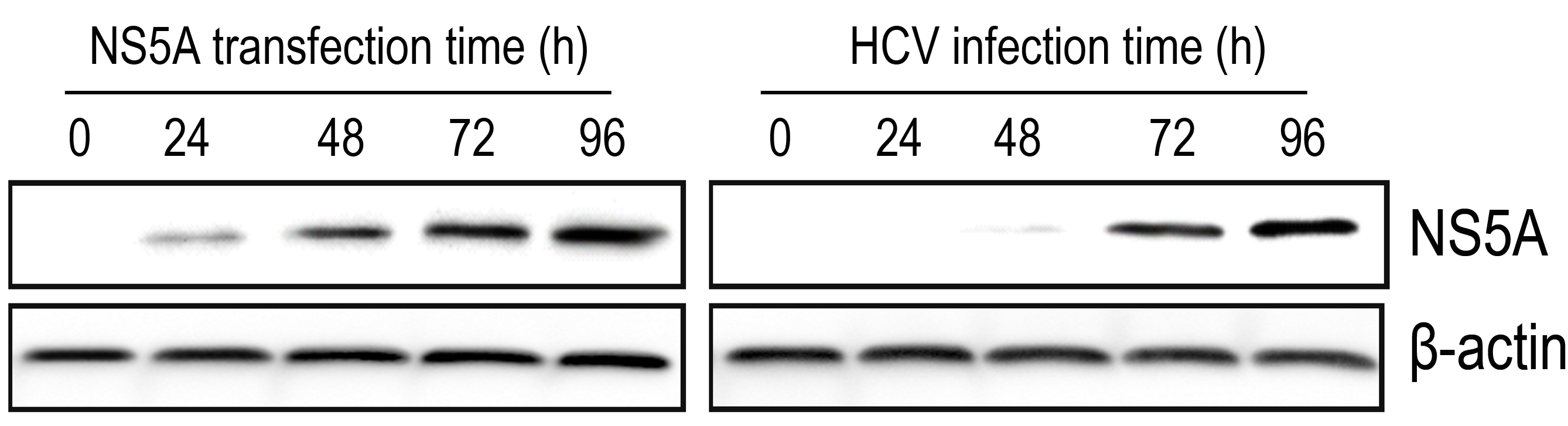

Supplement: Figure S9 — The expression level of NS5A protein during pCMV-NS5A transfection or HCV infection. Huh7 cells were transfected or infected with pCMV-NS5A (left panel) or HCV (MOI = 1) (right panel) for different times as indicated, respectively. The levels of NS5A were determined by Western blot and normalized to β-actin. Similar results were obtained in three independent experiments. (TIF) [file ppat.1003248.s009.tif]

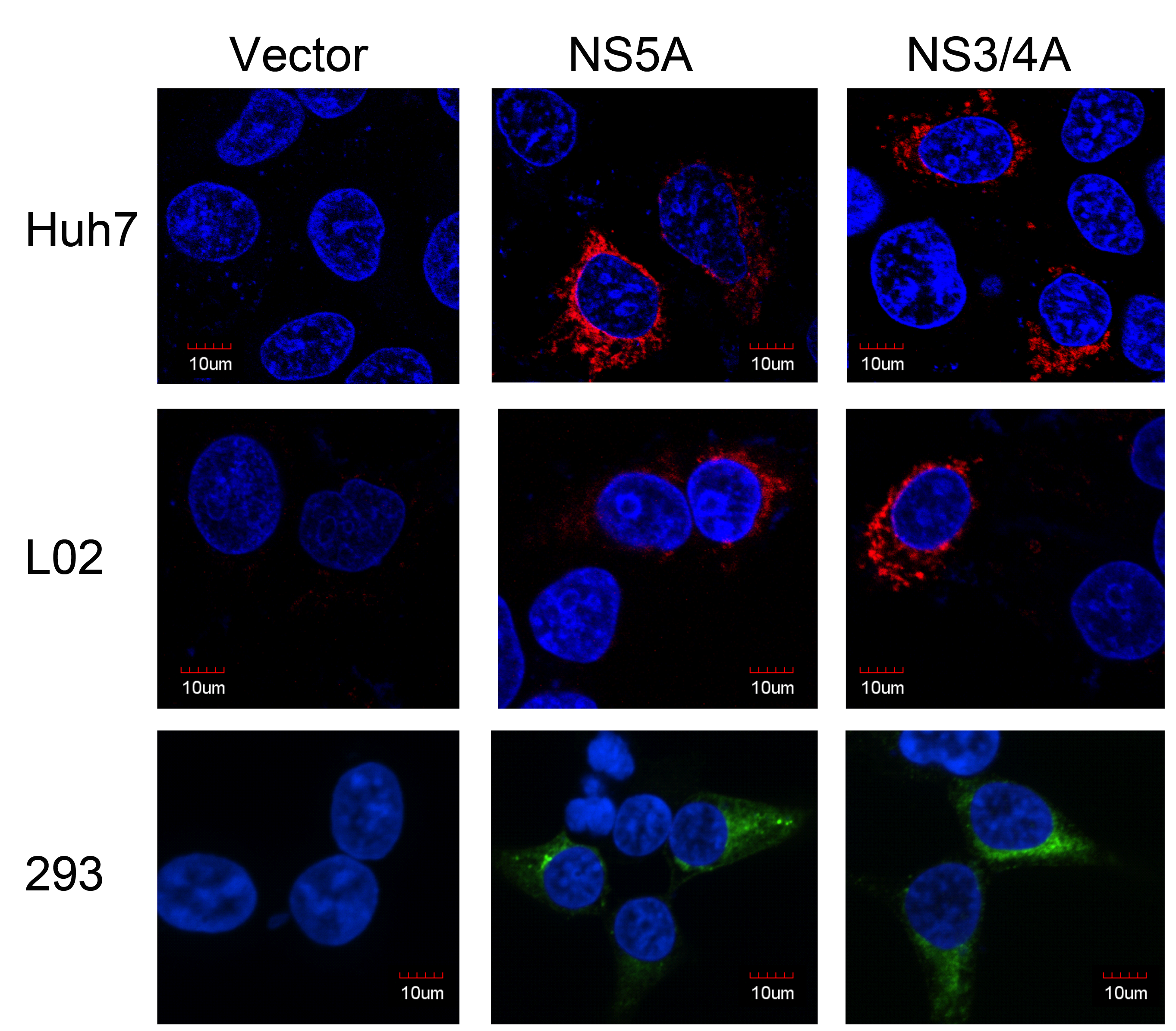

Supplement: Figure S10 — The localization of NS5A and NS3/4A protein in various cell types. Huh7 cells (upper panel) were transfected with pCMV-NS5A, pCMV-NS3/4A, or control vector as indicated, respectively, for 48 h. After fixation, the cells were immunostained with antibody for Flag. The nuclei were stained by DAPI. The L02 (middle panel) and 293 (lower panel) cells were transfected and treated as Huh7 cells. Similar results were obtained in three independent experiments. (TIF) [file ppat.1003248.s010.tif]
