# Supplementary material for: HCV-Induced miR-21 Contributes to Evasion of Host Immune System by Targeting MyD88 and IRAK1
Source: PLoS Pathog. 2013 Apr 25;9(4):e1003248. doi: 10.1371/journal.ppat.1003248 (PMC3635988; doi:10.1371/journal.ppat.1003248)
Supplement: Table S1 — Primers and oligonucleotide sequences in the study (DOC) [file ppat.1003248.s011.doc]

**Supplemental Table S1: Primers and oligonucleotide sequences in the study**

| **experiment** | **Primer Name** | **Sequence** |
| --- | --- | --- |
| olignucleotide | miR-21 mimics | 5’- UAGCUUAUCAGACUGAUGUUGA -3’ |
|  | miR-21 inhibitor  Ctrl mimics  Ctrl inhibitor | 5’- UCAACAUCAGUCUGAUAAGCUA -3’  5’- UUUGUACUACACAAAAGUACUG-3’  5’- CAGUACUUUUGUGUAGUACAAA-3’ |
| Report assays  (Mutated bases are indicated by underlines.) | f1_F | 5’- TAAGGTACCGAAGGAGCTCCGAGTACATAAA -3’ |
|  | f2_F | 5’- TAAGGTACCGGGAGGTGCCTCCCAAGTTT -3’ |
|  | f3_F | 5’- ACCGGTACCTCTGAGAAGAGGGGACAAGTCA -3’ |
|  | f4_F | 5’- TACGGTACCTAGTGGTGATAAATGTGGGACTTC -3’ |
|  | f1-f4_R  f6_F | 5’- AACTCGAGCTTACCACCTGGACTCAAAAGG -3’  5’- TTGTTCCTTTGTTAACTCCTGACTTCTGACTAG -3’ |
|  | f7_F | 5’- TTTGGATAAGGATAACACCCAGATTGTCCTAAT -3’ |
|  | f8_F | 5’- TGACACAAGCATAAACCCTTTCCTTATTAATTG -3’ |
|  | f9_F | 5’- TAATGCATTCTTTTTCCATAAGGATGACGCACA -3’ |
|  | f10_F | 5’- AAACCAGTTCTTACACCAACTAGTGGTGATAAA -3’ |
|  | f11_F | 5’- CATTTCCTTATTAATGCATTCAAACCAGTTCTT -3’ |
|  | f12_F | 5’- CTAATAAGGACTTAGACCGAGAAAGACCGCCCC -3’ |
|  | f14_F | 5’- CAAGTTTGCTAATGCTACCTTTTTGGATAAGGA -3’ |
| Primer extension | MyD88 3’UTR F: | 5’- ACTTGTGTGTGTGTTGAGTGGAAGA -3’ |
|  | MyD88 3’UTR R: | 5’- CTGGGTCATATGGTAATTCTGTTTG -3’ |
|  | IRAK1 3’UTR F: | 5’- TGTGTTCACCTGGGCAGATCCCCCA-3’ |
|  | IRAK1 3’UTR R: | 5’- TTATTGCAACATACGTTTTTATTAC -3’ |
| Primers for ChIP assay | mippR-21_F | 5’-CACCCCCGTCCCCTCCAA-3’ |
|  | mippR-21_R | 5’-GATCCCAGGTTGGCGGGC-3’ |
|  | GAPDH-promoter_F | 5’-CCACTGAAGCGGCAGAAACACA-3’ |
|  | GAPDH-promoter_R | 5’-TTGGTGGGGCTGAGGCTG-3’ |
