# Supplementary material for: HCV-Induced miR-21 Contributes to Evasion of Host Immune System by Targeting MyD88 and IRAK1
Source: PLoS Pathog. 2013 Apr 25;9(4):e1003248. doi: 10.1371/journal.ppat.1003248 (PMC3635988; doi:10.1371/journal.ppat.1003248)
Supplement: Table S2 — Primers used for qPCR. (DOC) [file ppat.1003248.s012.doc]

**Table S2:** Primers used for qPCR.

| MYD88 sense | 5'-TTCCAAGCCCACCACAAC-3' |
| --- | --- |
| MYD88 antisense | 5'-TCCCTCACCTGGAGAAGC-3' |
| IRAK1 sense | 5'-ACCGCAGATTATCATCAACC-3' |
| IRAK1 antisense | 5'-AGACTTACAGCCATACTTCACT-3' |
| IRAK4 sense | 5'-GCTGTATGTAGGGTGGAAAC-3 |
| IRAK4 antisense | 5'-TGCTGACAACTGGAAGGTAG-3' |
| TRAF6 sense | 5'-GCCCAGGCTGTTCATAGTTT-3' |
| TRAF6 antisense | 5'-CAAGGGAGGTGGCTGTCATA-3' |
| IRF-7 sense | 5'-TGGTCCTGGTGAAGCTGGAA-3' |
| IRF-7 antisense | 5'-GATGTCGTCATAGAGGCTGTTGG-3' |
| IFNAR1 sense | 5'-AAAATGGCAATGATAGG-3' |
| IFNAR1 antisense | 5'-CAGGCTGAGCAGAAGG-3' |
| IFNAR2 sense | 5'-AAATGCACCCTCCTTCC-3' |
| IFNAR2 antisense | 5'-GCCCTTAGCGAGACCTT-3' |
| IFN-α sense | 5'-TTTCTCCTGCCTGAAGGACAG-3' |
| IFN-α antisense | 5'-GCTCATGATTTCTGCTCTGACA-3' |
| HCV serotype sense | 5'-TCGTATGATACCCGATGCT-3' |
| HCV serotype antisense | 5'-GTTTGACCCTTGCTGTTGA-3' |
| GAPDH sense | 5'-AAGGCTGTGGGCAAGG-3' |
| GAPDH antisense | 5'-TGGAGGAGTGGGTGTCG-3' |
