# Supplementary material for: HCV-Induced miR-21 Contributes to Evasion of Host Immune System by Targeting MyD88 and IRAK1
Source: PLoS Pathog. 2013 Apr 25;9(4):e1003248. doi: 10.1371/journal.ppat.1003248 (PMC3635988; doi:10.1371/journal.ppat.1003248)
Supplement: Table S3 — Primers used for semiquantitative RT-PCR. (DOC) [file ppat.1003248.s013.doc]

**Table S3:** Primers used for semiquantitative RT-PCR.

| MYD88 sense | 5'-GCTGAGAAGCCTTTACAGGTG-3' |
| --- | --- |
| MYD88 antisense | 5'-CTGGGGCAATAGCAGATGAAG-3' |
| IRAK1 sense | 5'-CTGGAAGGCAGAAAAGTTGG-3' |
| IRAK1 antisense | 5'-TGTGACTCACGGCTGAACAC-3' |
| IRAK4 sense | 5'-AGGGAGGATTTGGAGTTGTA-3' |
| IRAK4 antisense | 5'-GAACCATTAGGCATGTAAC-3' |
| TRAF6 sense | 5'-ACAAACAAGCCACGGGAAAT-3' |
|  |  |
| TRAF6 antisense  IRF-7 sense  IRF-7 antisense | 5'-AGCAAAGCCCAAGAAAGTA-3'  5'-CGCGGCACTAACGACAGGCGAG-3'  5'-GCTGCCGTGCCCGGAATTCCAC-3' |
|  |  |
| β-actin sense | 5'-TGAAGTGTGACGTGGACATCCG-3' |
| β-actin antisense | 5'-GCTGTCACCTTCACCGTTCCAG-3' |
