# Supplementary material for: HCV-Induced miR-21 Contributes to Evasion of Host Immune System by Targeting MyD88 and IRAK1
Source: PLoS Pathog. 2013 Apr 25;9(4):e1003248. doi: 10.1371/journal.ppat.1003248 (PMC3635988; doi:10.1371/journal.ppat.1003248)
Supplement: Table S4 — Accession numbers. (DOC) [file ppat.1003248.s014.doc]

**Table S4: Accession numbers**

| **Gene** | **ID number** |
| --- | --- |
| MyD88 | ENSG00000172936 |
| IRAK1  IRAK4 | ENSG00000184216  ENSG00000198001 |
| TRAF6 | ENSG00000175104 |
| IRF7 | ENSG00000185507 |
| IFNAR1 | ENSG00000142166 |
| IFNAR2 | ENSG00000159110 |
| IFN-α | ENSG00000197919 |
| PKR | ENSG00000055332 |
| OAS | ENSG000000198732 |
| Mx | ENSG000000157601 |
| STAT1 | ENSG000000115415 |
| STAT2  TRIF  NS3/4A  NS5A  ERK  JNK  c-fos  c-jun  PKC-α  PKC-β1  PKCε  PKCζ  PKCδ  PKD1  PKD2  GAPDH | ENSG000000170581  223460864  164509879  111434539  ENSG000000100030  ENSG000000107643  ENSG000000170345  ENSG000000177606  ENSG000000154229  ENSG000000166501  ENSG000000171132  ENSG00000067606  ENSG00000027025  ENSG000000184304  ENSG000000105287  ENSG0000011640 |
